# NextGen sequencing reveals short double crossovers contribute disproportionately to genetic diversity in *Toxoplasma gondii*

**DOI:** 10.1186/1471-2164-15-1168

**Published:** 2014-12-23

**Authors:** Asis Khan, Jahangheer S Shaik, Michael Behnke, Qiuling Wang, Jitender P Dubey, Hernan A Lorenzi, James W Ajioka, Benjamin M Rosenthal, L David Sibley

**Affiliations:** Department of Molecular Microbiology, Washington University School of Medicine, Campus Box 8230, 660S, Euclid Ave., St. Louis, Mo 63110 USA; Animal Parasitic Disease Laboratory, Beltsville Agricultural Research Service, Agricultural Research Service, United States Department of Agriculture, Beltsville, MD 20705 USA; Department of Informatics, The J. Craig Venter Institute, 9704 Medical Center Drive, Rockville, MD 20850 USA; Department of Pathology, University of Cambridge, Cambridge, UK; Molecular Parasitology Unit, Laboratory for Parasitic Diseases, NIAID, NIH, Bethesda, MD 20892 USA

**Keywords:** Gene conversion, Genetic mapping, Meiotic drive, Mendelian inheritance, Double crossover, Gene conversion

## Abstract

**Background:**

*Toxoplasma gondii* is a widespread protozoan parasite of animals that causes zoonotic disease in humans. Three clonal variants predominate in North America and Europe, while South American strains are genetically diverse, and undergo more frequent recombination. All three northern clonal variants share a monomorphic version of chromosome Ia (ChrIa), which is also found in unrelated, but successful southern lineages. Although this pattern could reflect a selective advantage, it might also arise from non-Mendelian segregation during meiosis. To understand the inheritance of ChrIa, we performed a genetic cross between the northern clonal type 2 ME49 strain and a divergent southern type 10 strain called VAND, which harbors a divergent ChrIa.

**Results:**

NextGen sequencing of haploid F1 progeny was used to generate a genetic map revealing a low level of conventional recombination, with an unexpectedly high frequency of short, double crossovers. Notably, both the monomorphic and divergent versions of ChrIa were isolated with equal frequency. As well, ChrIa showed no evidence of being a sex chromosome, of harboring an inversion, or distorting patterns of segregation. Although VAND was unable to self fertilize in the cat, it underwent successful out-crossing with ME49 and hybrid survival was strongly associated with inheritance of ChrIII from ME49 and ChrIb from VAND.

**Conclusions:**

Our findings suggest that the successful spread of the monomorphic ChrIa in the wild has not been driven by meiotic drive or related processes, but rather is due to a fitness advantage. As well, the high frequency of short double crossovers is expected to greatly increase genetic diversity among progeny from genetic crosses, thereby providing an unexpected and likely important source of diversity.

**Electronic supplementary material:**

The online version of this article (doi:10.1186/1471-2164-15-1168) contains supplementary material, which is available to authorized users.

## Background

*Toxoplasma gondii* is a widespread parasite of animals that causes opportunistic infection in humans [1]. The parasite is transmitted by cats, which serve as the definitive host and shed infectious oocysts in their feces [2]. Oocysts undergo meiosis in the environment to form eight haploid sporozoites that are highly infectious to a variety of warm-blooded hosts [2]. Oocyst contamination of food or water leads to infection of a variety of intermediate hosts, including accidental infection of humans through food or waterborne transmission [3, 4]. Rodents are commonly infected in the wild and likely constitute a major natural host for transmission to cats, thus completing the cycle [2].

Studies on the population genetic structure of *T. gondii* have revealed complex patterns that differ among geographic regions [5]. Strains isolated in North America and Europe largely comprise three highly similar clonal lineages [6], with a fourth clonal variant found more commonly in wild animals in North America [7]. In contrast, strains in South America are genetically more divergent and appear to undergo more frequent recombination [8–10]. The close ancestry of the three northern clonal lineages has led to the hypothesis that they originated from a small number of genetic crosses in the past ~10,000 yrs [11, 12]. Subsequently, the three predominant clones expanded from this bottleneck to occupy many regions and hosts in North America and Europe, perhaps aided by human colonization [13]. In contrast, strains in South America date to a much older time period, and they have largely remained genetically isolated from those in the North [14]. Intriguingly, all members of the northern clonal isolates contain a monomorphic variant of ChrIa, consistent with their common ancestry [15]. In contrast to the rest of the genome where clonal lineages differ by 2-3% at the nucleotide level, ChrIa differs by < 1 in 10,000 bp among the clonal lineages. Surprisingly this monomorphic version of ChrIa is not restricted to northern isolates, but is also found in common South American lineages [8, 16]. This unusual pattern has led to the suggestion that ChrIa provides some fitness advantage in the wild [8, 16], although the basis for this success is not currently understood. Analysis of a large number of isolates indicates that recombination of ChrIa is infrequent with one of 4 distinct patterns being found repeatedly among different isolates: uniformly monomorphic, uniformly divergent, or chimeric with one end monomorphic and one end divergent (referred 3′ or 5′ chimeric) [8, 16]. Remarkably, these chimeric versions of ChrIa occur in multiple related clones that share the same pattern, suggesting they arose once and have since spread [8, 16]. Although this conserved pattern suggests an advantage to maintaining the monomorphic state of ChrIa, it might alternatively be preserved because natural recombination in many *T. gondii* lineages is rare [5].

Reduced recombination and non-Mendelian patterns of chromosomal inheritance have been described in other system where segregation of chromosomes can be influenced during meiosis by a variety of processes [17]. For example, sex chromosomes often show low levels of recombination as do some autosomal chromosomes such as Chr4 in *Drosophila*, which shows very low levels of polymorphism [18]. Selfish genetic elements have also been described that distort segregation at meiosis, due to a process called meiotic drive, or to processes that affect the inheritance of offspring due to segregation distortion [17]. Meiotic drive mechanisms typically affect the segregation of chromosomes during meiosis, while segregation distortion alters the production of gametes, and post-segregation distorters alter survival of offspring after meiosis [17]. Among the better studied examples of segregation distortion is the *Drosophila* Sd system, which consists of a drive locus called Sd and a responder locus called Rsp (other loci also modify these effects) [19]. The products of the Sd and Rsp loci are thought to interact to affect sperm development, favoring Rsp insensitive alleles in the presence of distortion activating Sd alleles [19]. The efficiency of segregation distortion systems can be enhanced by chromosome inversion, thus preventing breakup of the activating drive locus and the insensitive responder, which might otherwise be separated by recombination [17]. Distorter loci are also frequently found in sex chromosomes that lack recombination, again preserving their preferential allelic pairing [17]. Although such mechanisms of meiotic drive or segregation distortion have not been previously described in *T. gondii*, they might explain the unusual inheritance pattern of ChrIa.

The sexual cycle in domestic cats has been exploited to develop experimental genetics in *T. gondii* by crossing different strains and developing genetic linkage maps based on the segregation of genetic markers among haploid progeny [20]. Forward genetics based on quantitative trait locus mapping has been exploited to map the molecular basis of differences in virulence among representative clonal lineages in the mouse model [21]. Natural recombinants among the clonal variants of *T. gondii* in the wild are rare, and yet when such events do occur they can dramatically shape the subsequent population structure [22]. Consistent with this prediction, there is evidence that the forth clonal isolate in North America, a group called type 12, has undergone recent recombination with type 2 [7]. Additionally, hybrids are occasionally seen between the clonal types in North America [6, 23, 24]. However, there have been few examples of genetic recombination between distantly related isolates in the wild. This may simply reflect their geographic separation, but might also be due to a barrier to cross-fertilization. Experimental crosses have shown that the clonal lineages undergo self fertilization with equal frequency as out-crossing [25]. The extent to which this occurs in more divergent lineages has not been examined. All of the genetic crosses conducted to date have been between the relatively closely related clonal lineages, all of which harbor the monomorphic ChrIa. Therefore, it remains uncertain whether genetic crosses between more divergent and clonal lineages are experimentally possible, and to what extent ChrIa may influence the inheritance of progeny from such outcrosses.

In order to fairly consider alternatives for explaining the abundance of the monomorphic Chr1a in nature, we sought to examine its pattern of inheritance among the progeny of an experimental genetic cross. We crossed the clonal type 2 strain ME49 [26], which harbors a monomorphic version of ChrIa, with the type 10 strain VAND [27], which has a divergent version of ChrIa [8, 16]. Previous crosses have taken advantage of sequence polymorphisms detected by restriction fragment length polymorphisms (RFLPs) [20, 28, 29], or hybridization to microarray probes [30] to define allelic patterns in progeny from genetic crosses. These previous crosses of *T. gondii* identified both conventional single crossovers and what appeared to be short double crossovers [20], although the relative low precision of the genetic map made it impossible to define the precise size of these intervals. Such events may present either conventional double crossovers or gene conversion events, which have been described in a variety of systems [31].

Methods used to analyze previous genetic crosses of *T. gondii* were designed to distinguish polymorphisms between the closely related clonal lineages that are predominant in North America and Europe and as such they are not widely amenable to analyzing more diverse strains. To develop a more versatile and unbiased genetic mapping strategy that is also capable of defining precise intervals of recombination, we employed NextGen sequencing (NGS) to identify single nucleotide polymorphisms (SNPs) in the progeny of this new genetic cross. Our findings reveal that ChrIa does not influence the outcome of meiosis through a process of meiotic drive, incompatibility, or by acting as a sex chromosome. NGS-facilitated genetic mapping also uncovered an unexpectedly high frequency of small double crossovers that greatly increases the genetic diversity of recombinant clones, and may represent an important source of genetic variability in experimental crosses and in the wild.

## Results and discussion

### Generation of an outcross for *T. gondii*

**Figure 1:**
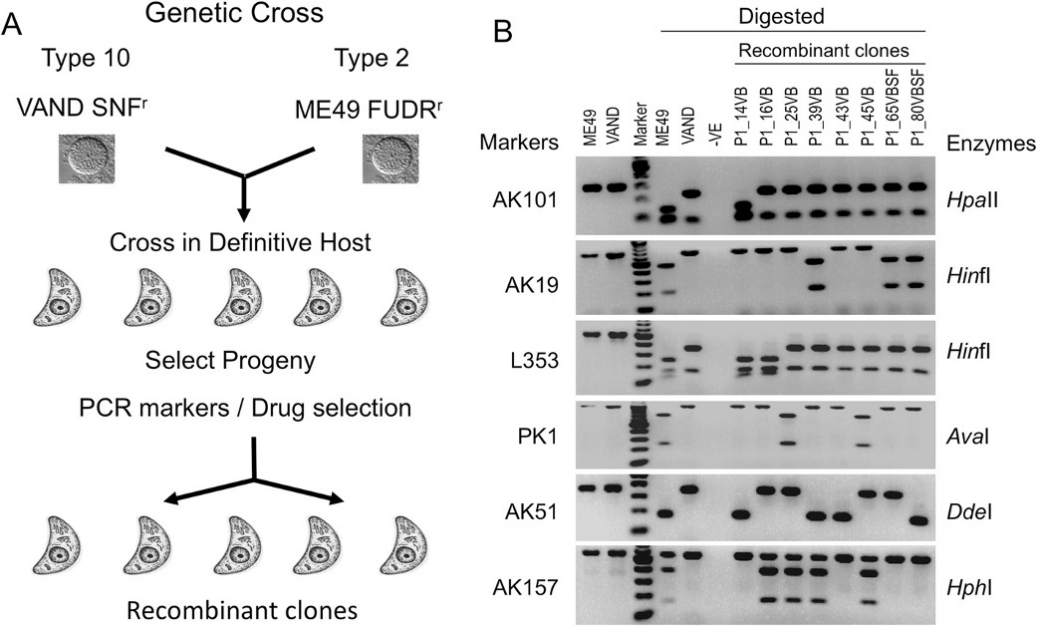
**Development of the genetic cross. A)** Schematic representation of the genetic cross between type 10 VAND and type 2 ME49 strains. Recombinant progeny were isolated either using dual drug selection or genotyping using eight RFLP makers located on separate chromosomes. **B)** Confirmation of recombinant progenies by PCR-RFLP markers. Markers are listed to the left, and restriction enzymes used in the digest to the right. V, VAND SNF^R^; M, ME49 FUDR^R^, clone names are indicated above the gel. –Ve, negative control.

We used a standard protocol to generate a genetic cross between the clonal type 2 ME49 strain and the exotic strain VAND, which is representative of a highly divergent South American genotype called haplotype 10 [14]. In brief, this involved generating chronic infections in mice and feeding infected mouse brains containing tissue cysts to susceptible cats and then collecting oocysts shed in the feces (Figure 1A). Before conducting the cross, we first passaged the parental strains through separate cats in order to collect fresh oocysts, as this process has been shown to increase fecundity and lead to greater success in crossing. We readily recovered oocysts of the ME49 fluorodeoxyribose – resistant (FUDR^r^) parent. However, we failed to recover oocysts from attempts to passage the VAND sinefungin –resistant (SNF^r^) parent in several independent cats. This defect was not due to an inability of VAND to form bradyzoites in chronically infected mice, but rather to a restriction in self-fertilization. Previous studies have shown that selfing occurs when a single cloned organism is used to initiate cat infection [32] and that it occurs as frequently as out-crossing when two different parasites are simultaneously co-inoculated in equal abundance [25]. Remarkably, when both strains in the present study were co-fed to a cat, viable oocysts were obtained that gave rise to both parental types, as well as recombinants. We initially isolated clones by growth in SNF and FUDR to estimate recombination frequency and to obtain doubly resistant progeny. The recombination frequency closely agreed with the theoretical expected frequency of 12.5% (assuming 50% out breeding and co-segregation of two unlinked markers [25]) (Table 1). We also screened clones using PCR-RFLP based makers, described previously [20], thereby easily identifying additional recombinant clones (Figure 1B). Using these markers we also identified what appeared to be both parental types from oocysts obtained from the cross. Following PCR typing of progeny, we chose 24 recombinant clones for further analysis, based on their initial genetic diversity (Additional file 1: Table S1).

**Table 1:** **Recombination frequency in the genetic cross between VAND and ME49**

Number of rarasites ^a^	No drugs ^b^	FUDR ^c^	SNF ^d^	FUDR and SNF ^e^	Calculations
10,000 parasites	96	70	80	10	
10,000 parasites	104	70	66	18	
10,000 parasites	120	72	90	13	
	106.67	70.67	78.67	13.67	Average
	12.22	1.15	12.05	4.04	S.D.
		12.81%	Recombination Frequency^d^		

^a^total number of parasites added to 6 well plate. Although the overall viability in this assay was low, the consistency across three replicates suggests that this did not affect the outcome.

^b^number of plaques formed in the absence of drugs at 7 days post-infection.

^c^number of plaques formed in the presence of fluorodeoxyuridine (FUDR, 3 × 10^-5^ M) at 7 days post-infection.

^d^number of plaques formed in the presence of synefungin (SNF, 3 × 10^-7^ M) at 7 days post-infection.

^e^number of plaques formed in the presence of both SNF and FUDR at 7 days post-infection.

^d^Calculation of recombination frequency = number of plaques formed in the presence of both drugs / the number of plaques formed in the absence of drugs × 100.

### Generation of a genetic linkage map

**Figure 2:**
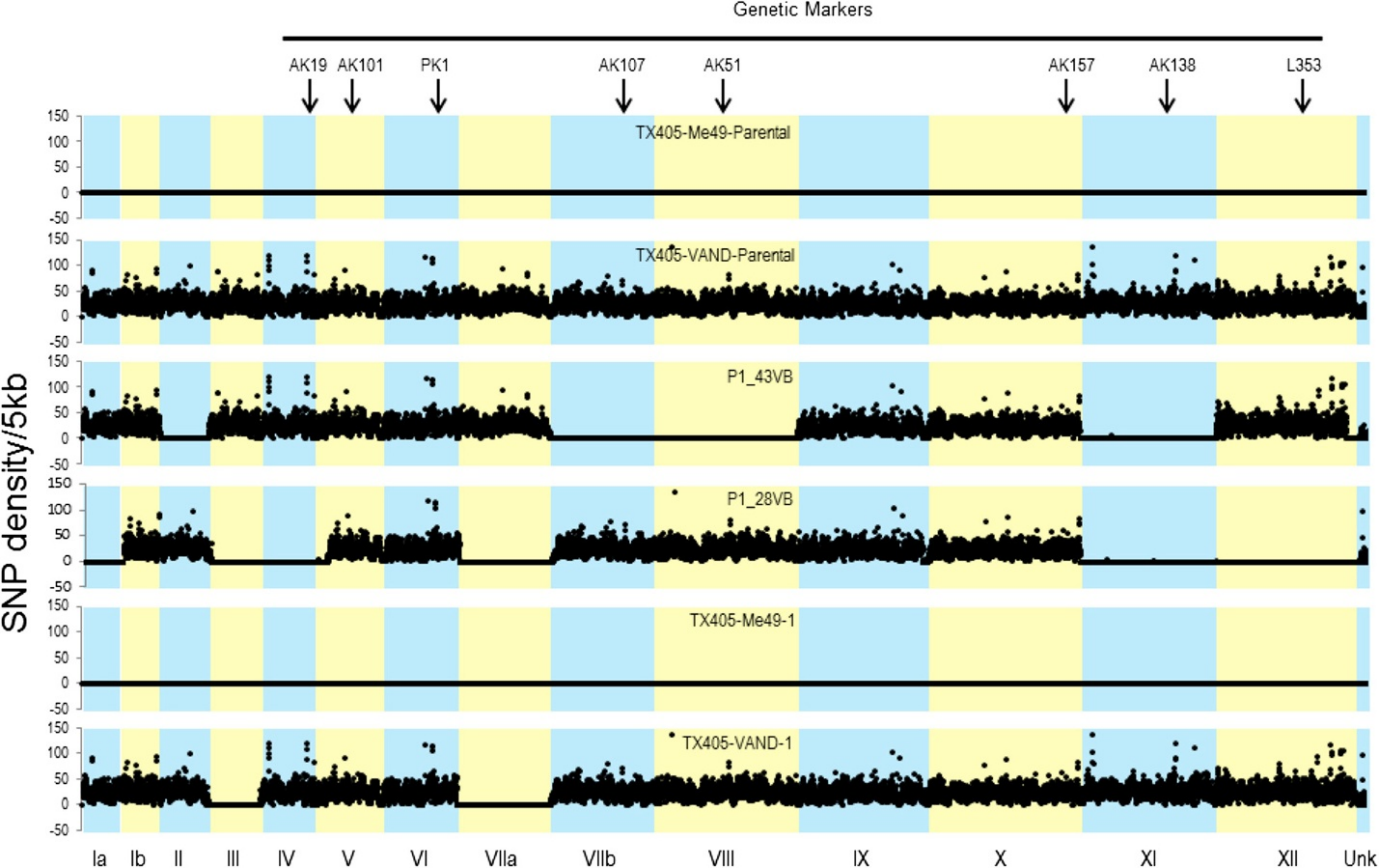
**SNP density plots of genome sequence reads from** ***T. gondii*** **clones compared against the ME49 reference genome.** The top two panels represent the ME49 and VAND parental lines. The middle two panels represent two recombinant clones that show mixed genotypes that match ME49 vs. VAND in different regions. The bottom two panels show a ME49 self-clone and VAND self-clone. X-axis indicates the relative sizes of 14 chromosomes of *T. gondii*. Y-axis indicates the number of SNPs per kb using a 5 kb sliding window. Top row indicates the location of 8 genetic markers used to determine the recombinant clones. Unk indicates unlinked and unmapped sequences.

Having shown that out-crossing between VAND and ME49 was experimentally feasible, we next explored the dynamics of chromosome segregation and recombination among progeny of the cross. In particular, we were interested in the inheritance patterns of Chr1, given the possibility that altered segregation might explain its abundance in the wild as an abundant haplotype. To generate a robust genetic map for this cross, we subjected both the parental strains and recombinant progeny to NGS using the Illumina platform. Sequence reads were mapped to the reference genomes for the ME49 and VAND strains, which were separately assembled and annotated as part of the *T. gondii* genomes consortium project (http://gsc.jcvi.org/projects/gsc/t_gondii/). Comparison of sequence reads from the recombinant progeny were used to identify SNPs and thereby determine the parental source of each variable position in the genome. A visual depiction of this read mapping is shown in Figure 2, where SNPs are plotted against the reference ME49 genome, resulting in few variant positions from the ME49 parental strain and ~ 30–50 SNPs/5 kb region for VAND. Recombinant progeny were easily identified as containing regions inherited from ME49 vs. VAND based on their SNP profile (Figure 2).

To more precisely identify SNPs and estimate the recombination intervals, we used a new utility called REDHORSE, which is described in an accompanying software methods paper [33]. In brief, the REDHORSE software identifies putative recombination breakpoints by evaluating SNPs in each progeny clone and comparing them to the genotype of the parents. By mapping the transition point between parental genotypes it identifies conventional recombinations, as well as double crossovers, which were defined here as two recombination events occurring within a 5 kb region. Most importantly, it also uses the physical position of polymorphisms in defining crossovers. REDHORSE was initially used to compare the ME49 and VAND reference genomes thereby identifying 499,470 informative SNPs that differ between the parental clones and for which there was sufficient data to genotype >70% of the progeny. REDHORSE detected a total of 79 distinct conventional crossovers among the 24 progeny based on these markers. The recombination breakpoints, together with buffer markers from the ends of the chromosomes were used to generate a genetic linkage map using MapDisto (Additional file 1: Figure S1). In addition, REDHORSE detected 59 positions where double crossovers between closely positioned markers, including some that occurred in multiple progeny (Additional file 1: Table S2); the nature and significance of these are discussed further below.

The strategy used here for generating linkage maps has several advantages over previous methods. Firstly, the availability of low cost NGS data allows whole genome polymorphism data to be rapidly acquired without prior knowledge of polymorphism, or the need to develop conventional probes such as microsatellites or RFLPs. Similar to other studies that have used NGS data to generate genetic maps for wheat [34], salmon [35], and apple [36], it was necessary to cull some of the sequence reads prior to generating alignments and mapping SNPs. This processing typically was required to accommodate repetitive or low complexity regions, which are inherently difficult to align with certainty. Here we have used very strict criteria to map reads (see methods), lending high confidence to all of the SNPs and markers included. Secondly, the precise cross over points can be mapped using transitions of genotypes defined by SNPs, including as described below, short double crossover regions. Thirdly, the map is readily expandable to accommodate new progeny. For example, here we have used only the informative crossover points among 24 progeny to generate the map. Additional SNPs between the parent strains occur in the merge data file generated by REDHORSE; however, since these differences are not informative among this set of progeny, they were not included in the map. If we were to add new progeny from this cross, it would be straightforward to identify new informative markers and expand the map accordingly. Finally, this new cross will foster further linkage analysis of variable phenotypic differences (i.e. growth, virulence, etc.,) between divergent and clonal lineages of *T. gondii*. However, prior to undertaking such studies, it is important to establish the basic properties of segregation and recombination in this outcross, as these parameters will affect the ability to map complex phenotypes.

### Out-crossing and selfing frequencies

Based on screening with PCR-RFLP markers, we identified 17 clones similar to the ME49 parent, 60 clones that appeared similar to the VAND parent, and 62 that were recombinants. The expected ratios should be ~ 1:2:1 based on 50% self-mating, although in this case we observed a higher frequency of apparent VAND self-clones, which as shown below may reflect the low density of markers used in this initial analysis. Regardless, the presence of apparent VAND genotypes contrasted sharply with the observation that VAND was unable to self-mate when fed to a cat alone. This disparity suggests two possible scenarios: VAND was unable to successfully self-mate in the cat, but was rescued by co-infection and underwent both self-mating and out-crossing or, 2) VAND was rescued by out-crossing, and despite their appearance as products of self-mating, the putative self-clones were in fact recombinants. The later scenario seemed unlikely because these self-clones had a VAND genotype at each of eight unlinked RFLP markers. Based on an expected 50% segregation of chromosomes from either parent, probability predicts that there would be 1 chance in 256 that actual recombinant clones would nonetheless have inherited the VAND genotype at each of the eight markers analyzed (1 in 2^8^).

To determine which of these two explanations was correct, we compared NGS of clones that appeared to inherit only ME49 (n = 6) or only VAND (n = 8) genotypes, respectively. All of the ME49 predicted self-clones showed a uniform ME49 genotype, confirming that each was indeed the product of self-mating (Figure 2 and Additional file 1: Figure S3). In contrast, six of the eight clones that initially appeared to be the products of VAND selfing were actually recombinants, bearing small genomic regions that matched ME49 (Figure 2 and Additional file 1: Figure S3). These regions corresponded to areas that lacked RFLP makers, and hence they were undetected in the initial screen. Only two of the sequenced clones had VAND sequence at all loci defined by SNPs (Additional file 1: Figure S3) consistent with the low fecundity of this strain when it was fed to cats alone.

Interestingly, 6 of 8 previously misclassified VAND recombinants inherited either all or part of ChrIII and ChrVIIa from ME49 (Additional file 1: Figure S3). The elevated frequency of inheritance of ChrVIIa from the ME49 parent was significantly different from the expected 50/50 ratio expected under random segregation (P ≤ 0.05), while the elevated frequency of inheritance of ChrIII was not quite significant, perhaps due to the small sample size (Additional file 1: Figure S3). These findings suggest that the low fecundity of VAND was rescued by inheritance of these regions of the ME49 genome. The pattern of co-inheritance of ChrIII and ChrVIIa from the ME49 parent was also seen in many recombinants clones from the cross, although in this case the inheritance of ChrIII was significant (*P* ≤ 0.05), while that of ChrVIIa was not (Additional file 1: Figure S2, S3). This pattern was strongest for ChrIII where 18 of 24 clones inherited all or part of this chromosome from ME49 (Additional file 1: Figure S2). Curiously, the opposite is seen for ChrIb, where all clones inherited part, or all, of this chromosome from the VAND parent, which was highly significantly different from the expected 50/50 ratio of segregation (*P* ≤ 0.0001, Additional file 1: Figure S2, S3). Neither of the drug resistance markers used to isolate recombinant resides on any of these chromosomes, suggesting this pattern was unrelated to the selection imposed by drug administration, and may instead reflect increased survival in the cat. Previous studies have shown that repeated passage of *T. gondii* can result in loss of cat transmission [37]. Although the basis for this defect is unknown, our study suggests that loss of self fertilization can be rescued by out-crossing, which might be an important means of generating increased diversity in the wild. Although we cannot precisely map the basis of the defect in VAND in the present cross, future backcross studies could be used to identify factors required for efficient transmission in the cat.

**Figure 3:**
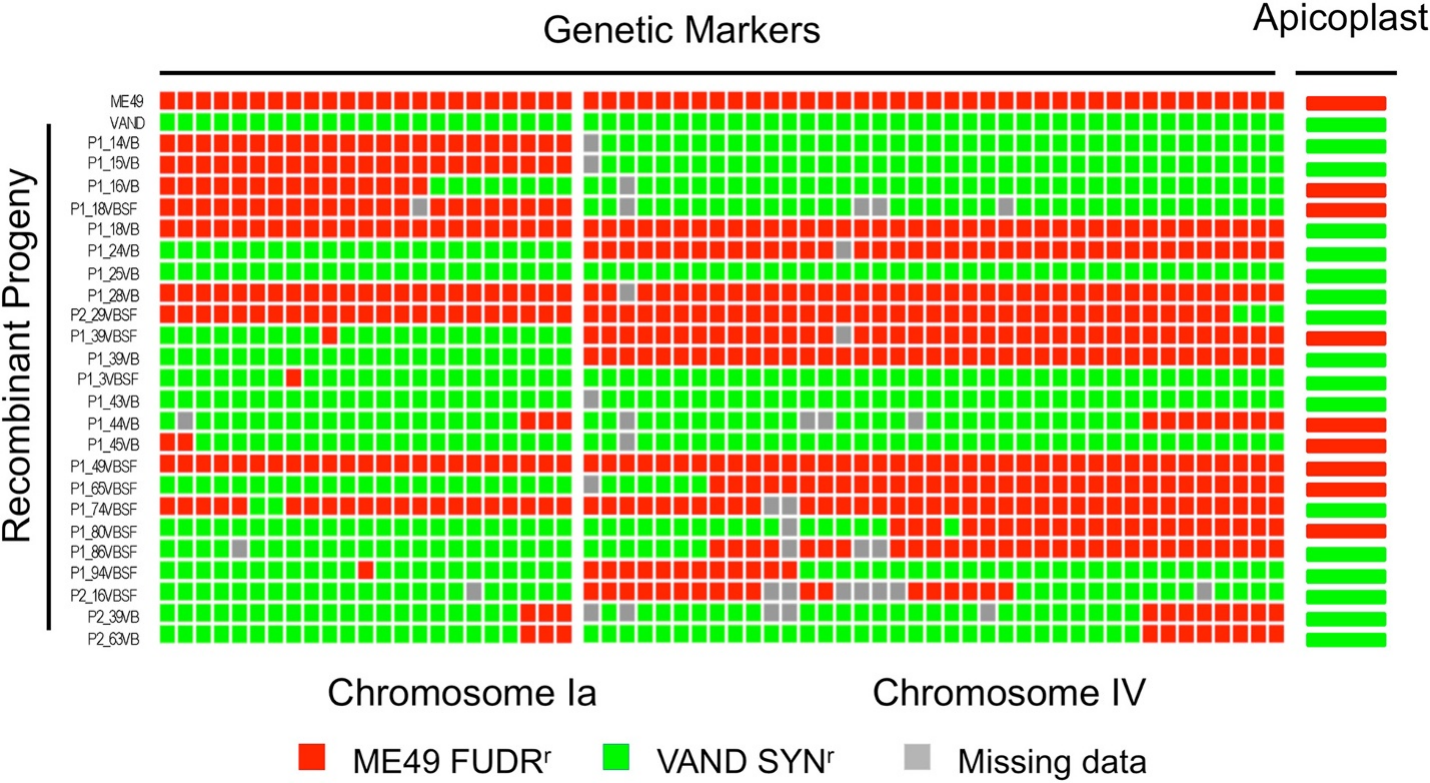
**Comparison of the genetic recombination rates on ChrIa vs. ChrV.** Progeny from the genetic cross between VAND SNF^r^ and ME49 FUDR^r^ were genotyped by whole genome sequencing to identify single nucleotide variants. Allelic patterns of the parental strains and 24 recombinant progeny are demonstrated at each SNP locus by solid boxes (red box, ME49 ChrIa and ChrIV, respectively; however, only SNPs that define crossovers in the recombinant progeny are shown here. Conventional single crossovers and short, double crossovers are seen by the alternating color block depicting genotype. Grey boxes indicate missing data. Profiles of the apicoplast genome are shown to the right.

It is uncertain why two clones of VAND that appear to be the result of pure selfing were obtained from the cross, when feeding of VAND tissue cysts alone to individual cats failed to result in detectable oocyst shedding. These two VAND self-clones might have resulted from the selfing-defect being incompletely penetrant, resulting in low level shedding of viable oocysts that were not readily detected in some cats. Alternatively, these VAND self-clones may have arisen from rescue of the defect by formation of a hybrid diploid zygote (i.e. by mating with a competent ME49 gamete), although the resulting genotype of these clones would require that all chromosomes were inherited from the VAND parent, without crossing over. Uniparental inheritance of chromosomes is commonly seen in genetic crosses of *T. gondii*[20], although the combined probability of this occurring on all chromosomes predicts this to be a rare event. Infertile mutants have previously been described in *Plasmodium berghei:* such mutants are typically defective in macro or microgametes, and thus can be rescued by crossing to wild type or compatible strains with compensating defects [38]. In contrast, the defect in VAND lies downstream of fertilization as it was capable of forming both functional micro and macrogametes, based on the genotype of the apicoplast, which is maternally inherited (Figure 3, right side).

### Patterns of chromosome inheritance

To examine the rates of recombination across individual chromosomes, we compared ChrIa with that of ChrIV, as they have similar genetic and physical sizes (Figure 3). Many chromosomes were inherited uniparentally without any apparent crossover and this pattern was similar on both Chr1a and ChrIV, consistent with previous genetic crosses using *T. gondii*[20]. We tested the frequency of inheritance of VAND vs. ME49 chromosomes and found that it did not differ significantly from the expected 50/50 ratio under the assumption of random segregation (Figure 3). As such, mechanisms such as meiotic drive or segregation distortion can be ruled out, as there was no evidence to suggest that the monomorphic ChrIa was preferred in the surviving progeny of the cross. Additionally, although only a minority of progeny showed evidence of intra-chromosomal recombination, this frequency was not significantly different from that observed in previous genetic crosses [20]. Double crossovers that occurred between closely adjacent markers were also seen in a number of progeny (Figure 3). By comparison with the inheritance of the maternally inherited apicoplast, ChrIa showed no evidence of being inherited as a sex-determining chromosome as the frequency of maternal inheritance differ not differ significantly from the expected 50/50 ratio (Figure 3, right column).

**Figure 4:**
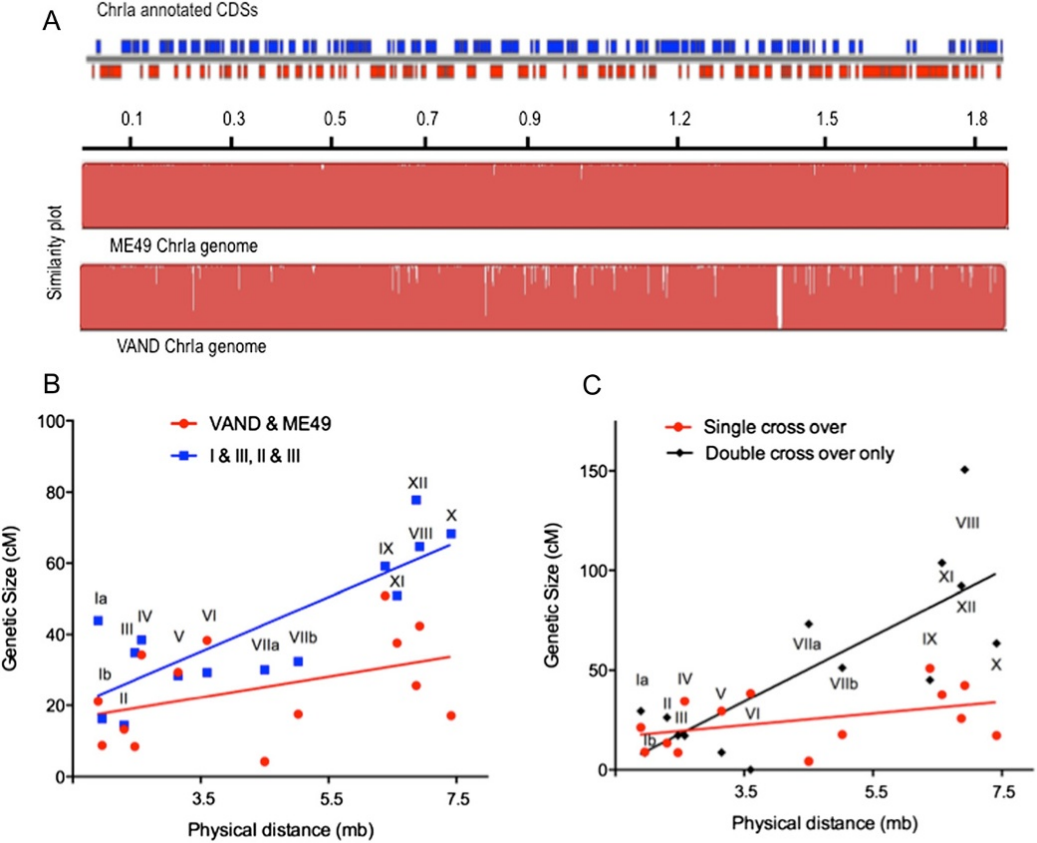
**Chr1a conservation and recombination among different chromosomes in the genetic cross. A)** Plot of the annotated coding sequences (CDS) on ChrIa (blue top strand, red, bottom strand) based on TOXODBv8 (top line). Beneath this are the similarity plots for the independent assemblies of the ME49 and VAND genomes constructed using Mauve (see methods). White bars reflect gaps in the assembly, typically denoted by “N”s in the reference sequence. **B)** Comparison of the relationship between physical size and recombination frequency in centimorgans (cM) for prior genetic crosses between clonal lineages (I &III, II&III) (blue symbols, r2 = 0.61)) and between VAND&ME49 (red symbols, r2 = 0.12). Comparison of the two curve fits revealed that the genetic distances of chromosomes (Y axis) of the prior genetic crosses (I&III, II&II) were significantly higher than that of the present VAND&ME49 cross (P ≤ 0.0005). **C)** Comparison of the relationship between physical size and recombination frequency in centimorgans (cM) for the VAND and ME49 genetic cross based on single conventional recombinations (red symbols) and double crossovers (black symbols). Chromosome numbers are indicated on the plot.

Another potential mechanism that might repress recombination across ChrIa in the wild would be if it contained an inversion that disfavored recombination due to potential disruption of genes that function as paired alleles across widely distant loci. To evaluate this possibility, we aligned the independently assembled whole genome sequences of ChrIa from ME49 and VAND and compared their coding capacity based on gene models and annotated genes in ToxoDB using Mauve [39]. There was no evidence for any major rearrangement although small gaps were observed due to differences in assemblies, likely the result of repeats or low complexity regions that are hard to assemble (Figure 4A). We also performed a similar analysis using NucMer [40] to compare either ChrIa, or the entire genomes of ME49 and VAND, again finding no major differences in the arrangement or content of the chromosomes (Additional file 1: Figure S4). Collectively, these data rule out several otherwise plausible mechanisms to explain the dynamics of ChrIa: neither alterations in meiosis, differential survival of gametes, status as a sex chromosome, nor a recombination-repressing inversion can account for its widespread success in the wild.

### Rates of recombination

To compare the rates of recombination across the 14 chromosomes, we plotted the physical size vs. genetic distance for prior crosses between the clonal lineages and compared this to the current ME49 × VAND cross (Figure 4B). In the case of the prior crosses, there was a linear relationship between the physical size of the chromosomes and genetic size (r2 = 0.61) (Figure 4B). In contrast, the linear regression fit for the present VAND × ME49 cross (r2 = 0.21) did not significantly differ from a slope of zero (Figure 4B). When the double crossovers from the present cross were analyzed, they showed a linear relationship with size, being especially frequent on larger chromosomes (although almost absent on V and VI) (Figure 4C). We compared the linear regression analyses shown in Figure 4B to determine if they were significantly different. Although the slope of the lines was not significant different, the previous crosses showed significantly higher genetic distances (Y axis) (*P* ≤ 0.0005) when compared to the VAND × ME49 cross, indicating that the recombination rate was lower in the present cross. In the present cross, individual chromosomes had genetic sizes that ranged from 4.18 cM (ChrVIIa) to 50.8 cM (ChrIX) and recombination was especially infrequent on ChrIb, ChrIII, ChrVIIa, and ChrXII (Figure 4B, Additional file 1: Figure S1). The total genetic size of the present genetic map was ~ 350 cM, whereas previous genetic maps between the clonal lineages indicated a combined size of ~ 590 cM [20]. This low rate of recombination may reflect the higher divergence of the VAND genome, compared to previous crosses between the type 2 ME49 and clonal types 1 [30] and 3 [20, 29]. Estimates of the ancestry of the clonal types suggest that a type 2-like strain was a parent for all three lineages [11], hence they may share greater compatibility for recombination. It is possible that the divergence of VAND represses recombination across certain chromosomes due to disruption of favorable pairing of alleles at distinct loci. However, when the double crossovers were combined with the singe events, the much larger genetic distances brought the combined rate to comparable levels (Figure 4C).

### Small double crossovers contribute to genetic diversity

**Figure 5:**
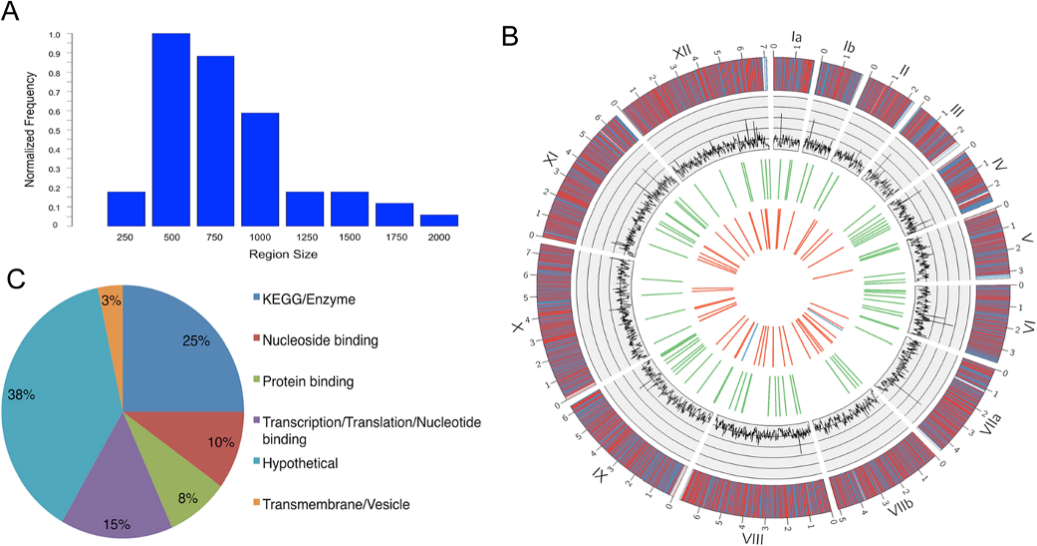
**Analysis of double crossovers. A)** Normalized histogram of double crossovers sizes. Double crossovers were defined as two consecutive crossovers that occur within 5,000 bp. The boundaries of the double crossovers were supported by at least six SNPs and the two boundaries were separated by at least five SNPs defining the crossover. The region sizes were counted in steps of 250 bp and normalized by highest count. **B)** Location of single and double crossovers depicted by Circos plot of the 14 chromosomes. The outside track plots the genes across the 14 chromosomes; top strand (blue) - bottom strand (red). The inside track plots the positions of the double crossovers that occurred in single progeny (red) vs. multiple progeny (blue). The second most inner track plots the positions of the conventional single crossovers (green). The second most outer track plots a histogram (black) of SNPs between VAND and ME49 per 10 kb rolling window, y-axis 0–500. **C)** Classification of the coding capacity of genes containing double crossovers. A total of 60 genes were identified that span a double crossover boundary. KEGG/Gene Ontology annotations obtained from ToxoDB.org were grouped based on functional categories. Chart represents percentage of the genes within each category.

The relative rarity of single conventional crossovers was offset by an elevated frequency of double crossovers. We analyzed the size of these and found that they spanned less than 5 kb, and typically were less than 1 kb (Figure 5A). By contrast, conventional crossovers typically only occurred over much longer distances. We plotted the distribution of all conventional single crossovers and double crossovers and found that they were relatively uniform across the chromosomes (Figure 5B). However, some chromosomes that had few conventional crossovers showed evidence of multiple small double crossovers (Figure 5B). Small double crossovers were previously seen in data based on micro-array hybridizations [30]; however, as they were not based on actual sequence data, it remained possible that they were artifacts. Additionally, small double crossovers between adjacent markers were previously reported in genetic crosses of *T. gondii* analyzed using RFLP-PCR based markers [20]. Because the physical distances between such markers varied, and SNP data between the markers was not available, it was not possible to precisely map the crossover points or accurately estimate their true sizes. Although prior studies indicated that double crossovers occur at a higher frequency than expected, they are still much less common than conventional crossovers among the clonal lineages [20]. In contrast, they were the more frequent events detected in the present cross (total conventional crossovers = 79, total double crossovers = 109).

We considered the possibility that in these apparent double crossovers might be artifacts of misaligned reads, especially in regions of low complexity and/or repetitive genomic regions. Although the stringency of the read mapping and allele calling parameters used here was designed to remove such events, we analyzed all the double crossovers for repeats or other features that might indicate less reliable regions. Even after strict filters were applied (see methods), we still found evidence for double crossovers including some specific regions that appeared to have undergone a double crossover in multiple progeny (Additional file 1: Table S2). To validate the double crossovers, we selected a subset of markers for PCR amplification and conventional Sanger sequencing. In 3 of 4 cases, we were able to amplify the region (one failed, presumably due to low complexity) and verify by Sanger sequencing that the double crossover occurred in the specific progeny (Additional file 1: Table S3). In one case, the same crossover was predicted to occur in three separate progeny in the same place, and this event was verified in one of these clones (Additional file 1: Table S3). Although the mechanism of such a process is presently not clear, it may be driven by low complexity or repeat regions. Indeed, when we relaxed the filters for 2× coverage, or regions that were found by RepeatMasker, we found far more evidence for such double crossovers appearing in multiple progeny (data not shown). At present, we cannot verify that these multiple events actually occur on a widespread basis due to the difficulty in unambiguously assigning reads from such regions. Nonetheless, the majority of the double crossovers that occur in one progeny do not occur in such repeat prone regions and they appear to be authentic (Additional file 1: Table S2, S3).

The nature of the events classified as double crossovers is not fully clear from the present studies as we are not able to obtain the separate products of a single meiosis and therefore cannot differentiate true double crossovers from gene conversions. Based on their short size, (generally less than 1,000 bp), these events would be classified as gene conversions in most systems [31]. Gene conversions typically occur by a double strand break repair process that occurs between regions of high homology [41]. In mammalian genomes, gene conversion events typically occur between paralogs, often involving a pseudo-gene conversion of an active gene, leading to genetic disease [31]. Additionally, interallelic gene conversion is thought to contribute to increased allelic diversity, for example in human blood group [42] and HLA haplotypes [43]. Although in some systems, motifs of alternating polypyrimidine or polypurine tracts, or simple repeats, have been associated with gene conversion [31], we did not observe such patterns in the sequences surrounding the double crossover events detected in *T. gondii*. The absence of such patterns may reflect the fact that we filtered repeat regions, and hence we may have discarded evidence for gene conversions occurring on a wider scale.

Regardless of the precise mechanism by which the short double crossovers are created in *T. gondii*, they are likely to be important for increasing genetic diversity following meiosis. For example of double crossover events detected in the current genetic cross, a majority occurred within genes (Additional file 1: Table S2). When we classified these gene using KEGG and Gene Ontology annotations, they occur in a wide variety of genes encoding proteins involved in transcription, translation, nucleotide metabolism, membrane trafficking, and protein-protein interactions (Figure 5C). Hence, the exchange of allelic variants by this process may be an important component of diversity generated by meiosis. This process has likely been overlooked in previous genetic crosses and population studies due to lower resolution of markers and the short nature of these double crossovers. In both types of studies, phenotypes are often broadly inferred from the genotype across haploblocks of the genome. This broad categorization may overlook functionally important differences that diverge from the genome as a whole [44] and this problem is magnified by the possibility of small blocks of recombination that elude detection. As such, future population and experimental genetic studies will be aided by genome-wide analysis of SNPs using the methods developed here.

### Evolutionary implications

On a broader evolutionary scale, the results of the present study provide insights about the evolutionary strategies of crossing vs. self-mating in natural populations of *T. gondii*. The efficiency of self-mating [25, 32], combined with the highly clonal population structure seen in regions such as North America [6, 45], might allow long range epistasis to develop, thereby suppressing intrachromosomal recombination. This pattern is seen in prior genetic crosses among the northern clonal lineages where chromosomes are often inherited uniparentally or with a single crossover [20]. The inheritance of large chromosomal blocks is also apparent in the ancestry of the northern clonal lineages in the wild [11]. In contrast to previously studied clonal lineages, VAND exhibits very low levels of self-mating, which might be due to extended laboratory passage, as reported previously [37]. However, absence of self-mating might also represent a stable evolutionary strategy in situations where opportunities for out crossing are high. One such location is South America where wild isolates also exhibit an absence of self-mating, even when tested at early passage [46]. This pattern is expected to prevail within a population structure with substantial out crossing, as evident in South America [8, 9]. The present experimental cross pits these two evolutionary strategies against each other. Here the inheritance of single intact chromosomes was favored, regardless of the parent of origin, supporting the idea of long-range epistasis. Interestingly, the observed low rate of conventional recombination was partially compensated for by the relatively greater frequency of double crossovers or gene conversion events, providing for increased genetic variability. Our studies make several interesting predictions for future testing: Out crosses between divergent strains with a history of recombination should show higher levels of conventional crossover. Clonal lineages should resist intrachromosomal recombination in situations where they undergo out crossing. Under such circumstances, short double crossovers or gene conversions may be the predominant means of introducing new genetic variation.

## Conclusions

We report here on the first outcross between a divergent strain of *T. gondii* and a conventional clonal isolate that has been subjected to previous genetic crosses. NGS-based genetic mapping revealed that although out-crossings result in lower levels of recombination, this feature might be compensated for by the frequent occurrence of small double crossovers (or gene conversions). These small double crossover events affected genes involved in diverse functions, thus serving as a previously unrecognized mechanism to increase diversity following genetic crosses. As they are small in size, such double crossovers would be missed in conventional mapping or associated studies, despite having potentially important biological influences. As such, these findings highlight the utility of high-resolution genetic maps based on whole genome sequencing. Our studies also revealed that the lack of self-mating by the divergent strain VAND was rescued by crossing with ME49 and this was associated with inheritance of specific chromosomes. We have recently shown that other exotic lineages from French Guiana have reduced fecundity in domestic cats, while domestic strains harboring the monomorphic Chr1a are more efficiently transmitted by this route [46]. Hence, out-crossing in the wild may be an important means of enhancing transmission of otherwise rare strains in the environment. Our studies rule out meiotic drive, segregation distortion, or status as a sex chromosome, as mechanisms to explain the paucity of recombination and very low polymorphism on ChrIa. By process of elimination, we conclude from these data that the success of ChrIa in the wild is likely to do enhanced transmission in natural hosts, survival in the environment, or demographic factors that affect its spread in anthropized environments. Combined with the ability of strains harboring this trait to cross-hybridize with rare variants in the wild, this may introduce new genes of considerable importance for pathogenicity into an otherwise fairly benign, yet common parasite.

## Methods

### Ethics statement

Laboratory mice were used for maintaining chronic infections of the parasite *T. gondii*. Mice were housed according to instructions in the “Guide to Care and Use of Laboratory Animals” under supervision of the veterinary staff in the Washington University Animal Care Facility. Protocols were approved by the Institutional Care Committee and are covered by animal welfare assurance number A-3381-01.

Domestic cats were used for genetic crosses as members of the cat family are the only known host for the sexual stages of *T. gondii.* Protocols were conducted in the laboratory of Dr. J. P. Dubey at the USDA in Beltsville MD. Dr. Dubey’s laboratory is approved for these procedures by USDA, ARS, Beltsville Agricultural Research Center Animal Care Committee (BAACUC) and are covered by animal welfare assurance number A4400-01.

### *Growth of T. gondii*strains and genotyping

*T. gondii* strains were cultured in monolayers of human foreskin fibroblast (HFF) cells maintained in Dulbecco’s modified Eagle’s medium supplemented with 10% fetal bovine serum, 2 mM glutamine, 20 mM HEPES (pH 7.5), and 10 μg/ml gentamicin (Invitrogen, Carlsbad, CA). Parasites were filtered after natural egress by passing through 3.0 μm polycarbonate filters to eliminate host cell debris and resuspended in phosphate buffered saline. PCR lysates were prepared by digesting with 10 μg/ml proteinase K (Sigma-Aldrich, St. Louis, MO) at 37°C for 1 h followed by 2 h incubation at 55°C and heat inactivation at 95°C for 15 min [14]. Lysates were used as template DNA for PCR amplification and RFLP analysis using a set of eight markers from the previously defined genetic map for *T. gondii*[20]. A list of progeny strains analyzed, and their phenotypes and genotypes at selected markers are provided in Additional file 1: Table S1.

### Isolation of recombinant progeny from a genetic cross

The ME49 strain used here was originally isolated from a sheep in North America [26] and It harbors a monomorphic version of Chr1a [15]. The VAND strain used here was originally isolated form a severe cases of human toxoplasmosis in French Guiana [27], and it harbors a divergent version of ChrIa [8, 16]. Drug resistant parental lines were generated for the type 2 ME49 and the type 10 VAND strains by chemical mutagenesis using *N*-ethyl-*N*-nitrosourea (200 μg/ml for 2 h at 37°C) (Sigma-Aldrich) followed by selection with sinefungin (SNF, 3 × 10^7^ M) or fluorodeoxyuridine (FUDR, 3 × 10^5^ M) as describe previously [20]. Resistant lines for VAND SNF^r^ and ME49 FUDR^r^ parasites were isolated by passage in drug on monolayers of HFF cells. In the case of ME49, a single plaque-purified clone (i.e. ME49 B7.21-E1) that was FUDR^r^ was further passaged in a cat (cross TX332) to derive the parental clone referred to as ME49 FUDR^r^. The pool of VAND SNF^r^ parasites was used without sub-cloning. Outbred CD-1 mice (JAX laboratories, Bar Harbor, MA) were infected intraperitoneally with these two parental drug resistant lines to develop chronic infections. Mice infected with VAND SNF^R^ strain, which is naturally more virulent, were maintained by treatment with 0.5 mg/ml of sulfadiazine (Sigma-Aldrich) in drinking water. One month post infection, tissue cysts were harvested by homogenizing infected mouse brains and the presence of tissue cysts was confirmed by staining with fluorescent *Dolichos biflorus* lectin [47]. Naïve specific pathogen free cats were co-fed tissue cysts to generate recombinant progeny. To generate the cross, a single cat was co-fed tissue cysts from the ME49 FUDR^r^ and VAND SNF^r^ parental lines. In parallel, several cats were challenged with VAND SNF^r^ tissue cysts alone, although these animals did not shed oocysts. Oocysts were purified by sucrose flotation, sporulated as described previously [48]. Oocysts were induced to hatch by physically braking the oocyst wall with glass beads and treatment with 5% sodium taurodeoxycholate (Sigma-Aldrich) in Hanks’ balanced salt solution containing 10 mM HEPES and 1 mM EGTA for 10 min at 37°C. Sporozoites were used to infect HFF monolayers and progeny isolated by limited dilution. The recombination frequency was determined by plating the dilutions of parasites in the presence of no drug, single drug or double drug combinations and determining the frequency of plaques on monolayers of HFF cells. Recombinant clones were selected in one of two ways: 1) 23 clones were obtained by limiting dilution after growth in both SNF and FUDR, or 2) 24 clones were selected randomly and genotyped using eight independent RFLP markers. Based on genotyping at 10 independent loci, 24 clones were selected based on their genetic diversity (Additional file 1: Table S1).

### Alignment of whole genomes to detect inversions or rearrangements

Under a separate project conducted at the J. Craig Venter Institute, the complete genome sequences of ME49 and VAND were obtained by the *T. gondii* genomes consortium (http://gsc.jcvi.org/projects/gsc/t_gondii/). Genomes were sequenced to 26.6× (ME49) and 39× (VAND) fold coverage using a combination of Sanger, 454 GS FLX Titanium and Illumina sequencing technologies. The ME49 and VAND genomes were independently assembled with Celera Assembler [49] and Newbler [50] and structural annotations were carried out with Evidence Modeler (EVM) [51]. The assembled genomes together with their annotations have been deposited in GenBank (ABPA02000000 and AEYJ00000000.2) and in ToxoDB (http://www.toxodb.org).

To examine potential rearrangements, the separately assembled whole genomes of ME49 and VAND (ToxoDB V.8.0) were aligned using Mauve 2.3.1 [39] with default minimum Locally Collinear Blocks (LCBs) using the following parameters: Match seed weight, 15; Aligner, Muscle 3.6; Minimum island size, 50; Maximum backbone gap size, 50; Minimum backbone size, 50. The Nucmer utility from MUMmer 3.0 [40] was also used to compare the independently assembled genomes of ME49 and VAND using the following default parameters. Nucmer alignments were visualized with the MUMmer tool Mummerplot.

### Developing a high-resolution genetic linkage map

To develop a genetic linkage map, we used polymorphism information obtained by comparing the while genomes sequences of the parental strains ME49 and VAND, as described above. We then compared whole genome sequences for select progeny clones from the genetic cross to define the inheritance of genomic regions based on SNPs, as defined below.

Recombinant progeny were selected for analysis based on the segregation patterns of the 10 RFLP markers to encompass a wide diversity of segregation patterns. Progeny were genotyped by paired-end genome sequencing (~15× coverage) using the Illumina HiSeq 2000 system (Genome Technology Access Center (GTAC), Washington University School of Medicine). Genomic sequences generated in this project were deposited in the SRA of NCBI under the following BioProject ID: PRJNA258152 and can be found at the following link (http://www.ncbi.nlm.nih.gov/bioproject/258152). The raw reads from the parental lines as well as hybrids were aligned to the ME49 genome v 8.0 by CLC genomics (http://www.clcbio.com) using the following parameters- Mismatch cost: 3, Insertion cost: 3, Deletion cost: 3, Length fraction: 0.9, Similarity fraction: 0.8 and by using Global alignment. Since the ME49 genome is haploid, we extracted the alleles with frequency greater than or equal to 80% and having a minimum coverage of 5 using REDHORSE [33]. Loci that did not meet these criteria were tagged as missing data. SNPs between the parental lines were used to define the inheritance of alleles in the hybrids. We identified a total of 532,949 SNPs in VAND and 1,821 SNPs in ME49 to compare raw reads to the reference ME49 genome in ToxoDB using REDHORSE [33]. The 301 SNPs that were common across both the parental lines were filtered out as they represent loci where both parents are different from the reference genome but are not different with respect to each other. This left us with 533,250 SNP loci where both parental lines were different from each other. The loci with more than 3 SNPs in 7 bp window represent noisy loci and were filtered out resulting in 520,013 SNPs. The SNPs that fell into regions with coverage greater than or equal to 3 times the baseline (mean coverage across the chromosome) were eliminated. A “merged allele” file was generated by extracting allele composition of hybrids as well as parental lines at these SNV loci. The “merged allele” file contains not only the contig information of each of the samples but also includes the chromosome and position information to accurately pinpoint crossover. The “merged allele” file was subjected to further filtering as follows: 1) a global filter eliminated the loci where more than 30% of the samples had missing data, 2) loci where either of the parents had missing data were filtered out and 3) multi-allelic loci were eliminated. This resulted in a final list of 499,470 loci that were used to detect conventional crossovers as well as double crossovers using REDHORSE.

REDHORSE was used to compare the sequence reads from each progeny to the respective parental genotypes. Crossovers were defined by changes in the genotype based on a window of 10 consecutive markers that define the break point for a conventional crossover (i.e. 4 consecutive VAND SNPs followed by 6 or more consecutive ME49 SNPs define a conventional crossover as in VVVVMMMMMM or vice versa). The double crossovers were defined as more than two break points occurring in 5 kb region. The double crossovers must include 5 or more SNPs that differ from the two break points within 5 kb region (i.e. VVVVVMMMMMVVVVV or vice versa). The double crossover regions were further tested by a couple of criteria to determine if they represent repeats in which case they were filtered out: 1) regions tagged by RepeatMasker (http://www.repeatmasker.org/) and 2) 500 bp segments around the crossover regions blasted against the ME49 genome that have multiple hits.

Using the “merged allele” file that contains the allelic composition of the samples at these crossover regions and using anchor loci from the beginning of the chromosomes, genetic maps were drawn using the conventional single crossovers using MapDisto [52] (Additional file 1: Figure S1).

To determine the apicoplast genome for the sequenced strains, we aligned Illumina reads for each of the progeny to the published (GenBank: U87145.2 or NC_001799.1) complete RH (type 1) apicoplast genome.

### Analysis of double crossover regions

Double crossovers were grouped based on size into bins of 250 bp and plotted based on normalized frequency. Double crossovers were analyzed based on their position in the genome to determine if they fell within or near (within 1,000 bp) coding region (ToxoDB.org) (Additional file 1: Table S2). Genomic positions for genes containing double crossovers were obtained from ToxoDB.org. The location of all annotated genes, single and double crossover events (excluding those that had multiple hits to the genome (Additional file 1: Table S2), and SNPs, were graphed using the software Circos [53]. Genes that overlapped double crossovers were identified and KEGG/Gene Ontology annotations were obtained from ToxoDB.org. Gene annotations were grouped into several high level functional categories and graphed using Excel.

### Validation of double crossovers

Four predicted double crossovers were chosen for PCR amplification and direct sequencing. Primers were designed to amplify the double crossover regions (Additional file 1: Table S3) and used in a Q5 High-Fidelity DNA Polymerase PCR (NEB, Inc., Ipswich, MA) with genomic lysate of the respective progeny being tested. Primers designed to one double crossover, chromosome VIII at position 5175014–5176112 (Additional file 1: Table S3) did not amplify a specific band by PCR, likely due to low-complexity sequence surrounding the double crossover. The remaining three products yielded a single discrete band. PCR products were cleaned using a QIAquick PCR Purification Kit (Qiagen, Valencia, CA) and sent to GeneWiz Inc., (Plainfield, NY) for Sanger sequencing. Sequence alignments were examined in ClustalW [54] to compare genotypes of the parental and progeny clones.

### Statistics

The frequency of chromosomal inheritance among the progeny was tested to determine if segregation differed from the expected 50/50 ratio of parental types using a Binomial test, two tailed, where *P* ≤ 0.05 was considered significant (Prism, GraphPad). The frequency of chromosomes containing one or more conventional crossovers, vs. those that were inherited uniparentally was tested compared to the expected frequency of crossing over based on previous crosses [20], using a Binomial test, two tailed, where *P* ≤ 0.05 was considered significant (Prism, GraphPad). The relationship between genetic distance and physical size was estimated based on linear regression analysis and comparisons of the slope vs. intercept (Y values) were compared using analysis of covariance where *P* ≤ 0.05 was considered significant (Prism, GraphPad).

### Availability of supporting data

Sequence reads for the progeny and parental strains studied here were deposited to the NCBI short read archive and are available at the following link http://www.ncbi.nlm.nih.gov/bioproject/258152.

## Electronic supplementary material

Additional file 1: **The following data are available with the online version of this paper.** Figure S1 contains genetic linkage maps of the chromosomes from the VAND and ME49 cross. Figure S2 contains the SNP plots from the recombinant progeny mapped against the reference ME49 genotype. Figure S3 contains the SNP plots of the ME49and VAND self-clones mapped against the reference ME49 genotype. Figure S4 contains alignment plots for comparison of Chr1a and the whole genomes of ME49 and VAND. Table S1 contains a list of recombinant progeny used in this study and their RFLP based genotypes at select loci. Table S2 contains annotated genes that contain double crossovers. Table S3 contains the genotype information for validated double crossover regions. (PDF 7 MB)

## Abbreviations

Chr: Chromosome
cM: Centimorgans
DC: Double crossover
FUDR: Flurodeoxyuridine
HFF: Human foreskin fibroblasts
NGS: NextGen sequencing
PCR: Polymerase chain reaction
RFLP: Restriction fragment length polymorphism
SNF: Sinefungin
SNP: Single nucleotide polymorphism.
