# Supplementary material for: NextGen sequencing reveals short double crossovers contribute disproportionately to genetic diversity in Toxoplasma gondii
Source: BMC Genomics. 2014 Dec 23;15(1):1168. doi: 10.1186/1471-2164-15-1168 (PMC4326188; doi:10.1186/1471-2164-15-1168)
Supplement: Supplementary file 1 — Additional file 1: The following data are available with the online version of this paper. Figure S1 contains genetic linkage maps of the chromosomes from the VAND and ME49 cross. Figure S2 contains the SNP plots from the recombinant progeny mapped against the reference ME49 genotype. Figure S3 contains the SNP plots of the ME49and VAND self-clones mapped against the reference ME49 genotype. Figure S4 contains alignment plots for comparison of Chr1a and the whole genomes of ME49 and VAND. Table S1 contains a list of recombinant progeny used in this study and their RFLP based genotypes at select loci. Table S2 contains annotated genes that contain double crossovers. Table S3 contains the genotype information for validated double crossover regions. (PDF 7 MB) [file 12864_2014_6991_MOESM1_ESM.pdf]

Supplemental Figure Legends

Fig. S1 Genetic linkage maps of *T. gondii* chromosomes using F1 progeny from the ME49 and VAND genetic cross. All the recombination points were identified by whole genome sequencing of recombinant progeny from the ME49 and VAND cross. Linkage maps were constructed using the recombination points as genetic markers. Relatedness between markers and linkage groups were determined by following criteria; minimum LOD score 3, d max 0.3, and physical location of each markers in ToxoDB.org. Genetic distances between markers are indicated on the left side in centimorgans (cM), marker names are listed on the right side.

Fig. S2 SNP density plots used to infer the inheritance of chromosomal regions among the F1 recombinant progeny of the ME49 and VAND cross. X-axis indicates the size of 14 chromosomes of *T. gondii*. Y-axis indicates the number of SNPs per 5 kb sliding window. Top row indicates the location of 8 genetic markers used to identify recombinant clones. Unk indicates unlinked and unmapped sequences. Red boxes mark chromosomes that show segregation distortion patterns that differ from the expected 50/50 ratio. ChrIb shows significantly higher inheritance of VAND ($P \leq 0.0001$) while ChrIII shows significantly higher inheritance of ME49 ($P \leq 0.05$) genotypes compared to the expected 50/50 ratio. The inheritance patterns of the other chromosomes were not significantly different from the expected 50/50 ratio. Binomial test, two-tailed.

Fig. S3 SNP density plots used to infer the inheritance of chromosomal regions among the apparent “self” clones from the ME49 and VAND cross. The X-axis indicates the size of 14 chromosomes of *T. gondii*. The Y-axis indicates the number of SNPs per 5 kb sliding window. The top row indicates the location of 8 genetic markers used to identify recombinant clones. Unk indicates unlinked and unmapped sequences. Red boxes mark chromosomes that show segregation distortion patterns that differ from the expected 50/50 ratio. The increased inheritance of the ME49 genotype for ChrIII was not statistically significant, likely due to the small sample size. However, the increased inheritance of the predominantly ME49 genotype for ChrVIIa was statistically significant ($P \leq 0.05$). Binomial test, two-tailed.

Fig. S4 Dot-plot representation of Nucmer whole genome alignments between *T. gondii* ME49 and VAND assemblies. A) Dot-plot detail of a Nucmer alignment between ChrIa from VAND and ME49 strains. ChrIa in VAND is comprised of two major scaffolds that comprise 99.5% of the entire sequence and they are shown here next to the plot of the combined assembly. Our analysis is not able to detect inversion breakpoints that might fall in-between scaffolds, although any such rearrangement is predicted to be very small. Chromosome positions in base pairs are depicted underneath and on the left of the figure. B) Whole genome alignment between VAND and ME49 assembly sequences. Red lines, alignments between two forward strands; blue lines, alignment between forward and reverse strands. Although small (generally less than 500 bp) rearrangements were detected on some chromosomes (seen as red and blue dots that fall of the central diagonal) none of these fall on ChrIa.

Fig. S1

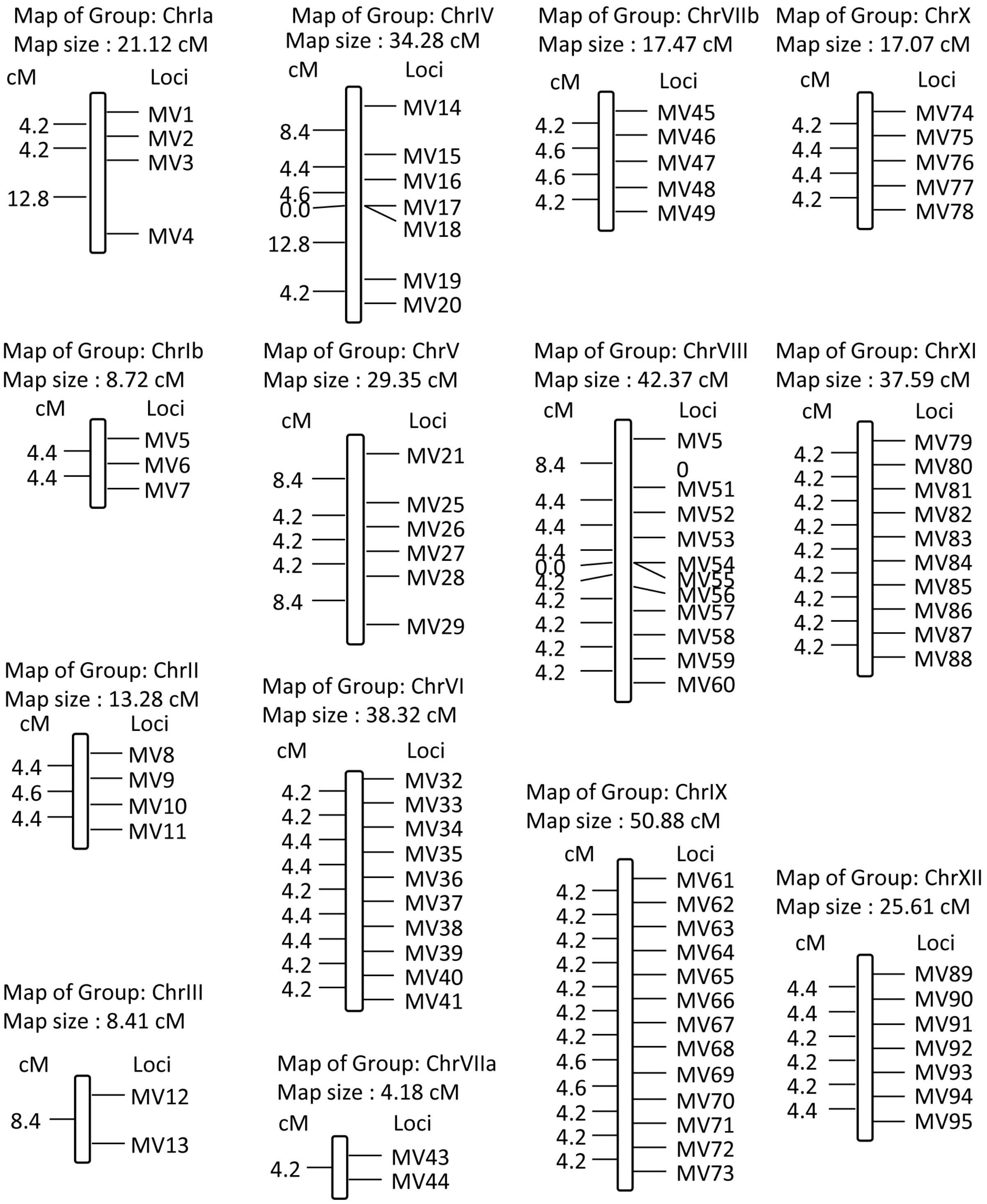

Map of Group: ChrIa
Map size : 21.12 cM
cM
Loci
4.2
4.2
12.8
MV1
MV2
MV3
MV4
Map of Group: ChrIV
Map size : 34.28 cM
cM
Loci
8.4
4.4
4.6
0.0
12.8
4.2
MV14
MV15
MV16
MV17
MV18
MV19
MV20
Map of Group: ChrVIIb
Map size : 17.47 cM
cM
Loci
4.2
4.6
4.6
4.2
MV45
MV46
MV47
MV48
MV49
Map of Group: ChrX
Map size : 17.07 cM
cM
Loci
4.2
4.4
4.4
4.2
MV74
MV75
MV76
MV77
MV78
Map of Group: ChrIb
Map size : 8.72 cM
cM
Loci
4.4
4.4
MV5
MV6
MV7
Map of Group: ChrV
Map size : 29.35 cM
cM
Loci
8.4
4.2
4.2
4.2
8.4
MV21
MV25
MV26
MV27
MV28
MV29
Map of Group: ChrVIII
Map size : 42.37 cM
cM
Loci
8.4
4.4
4.4
4.4
0.0
4.2
4.2
4.2
4.2
4.2
MV5
0
MV51
MV52
MV53
MV54
MV55
MV56
MV57
MV58
MV59
MV60
Map of Group: ChrXI
Map size : 37.59 cM
cM
Loci
4.2
4.2
4.2
4.2
4.2
4.2
4.2
4.2
4.2
MV79
MV80
MV81
MV82
MV83
MV84
MV85
MV86
MV87
MV88
Map of Group: ChrII
Map size : 13.28 cM
cM
Loci
4.4
4.6
4.4
MV8
MV9
MV10
MV11
Map of Group: ChrVI
Map size : 38.32 cM
cM
Loci
4.2
4.2
4.4
4.4
4.2
4.4
4.4
4.2
4.2
MV32
MV33
MV34
MV35
MV36
MV37
MV38
MV39
MV40
MV41
Map of Group: ChrIX
Map size : 50.88 cM
cM
Loci
4.2
4.2
4.2
4.2
4.2
4.2
4.2
4.6
4.6
4.2
4.2
4.2
MV61
MV62
MV63
MV64
MV65
MV66
MV67
MV68
MV69
MV70
MV71
MV72
MV73
Map of Group: ChrXII
Map size : 25.61 cM
cM
Loci
4.4
4.4
4.2
4.2
4.2
4.4
MV89
MV90
MV91
MV92
MV93
MV94
MV95
Map of Group: ChrIII
Map size : 8.41 cM
cM
Loci
8.4
MV12
MV13
Map of Group: ChrVIIa
Map size : 4.18 cM
cM
Loci
4.2
MV43
MV44

Fig. S2

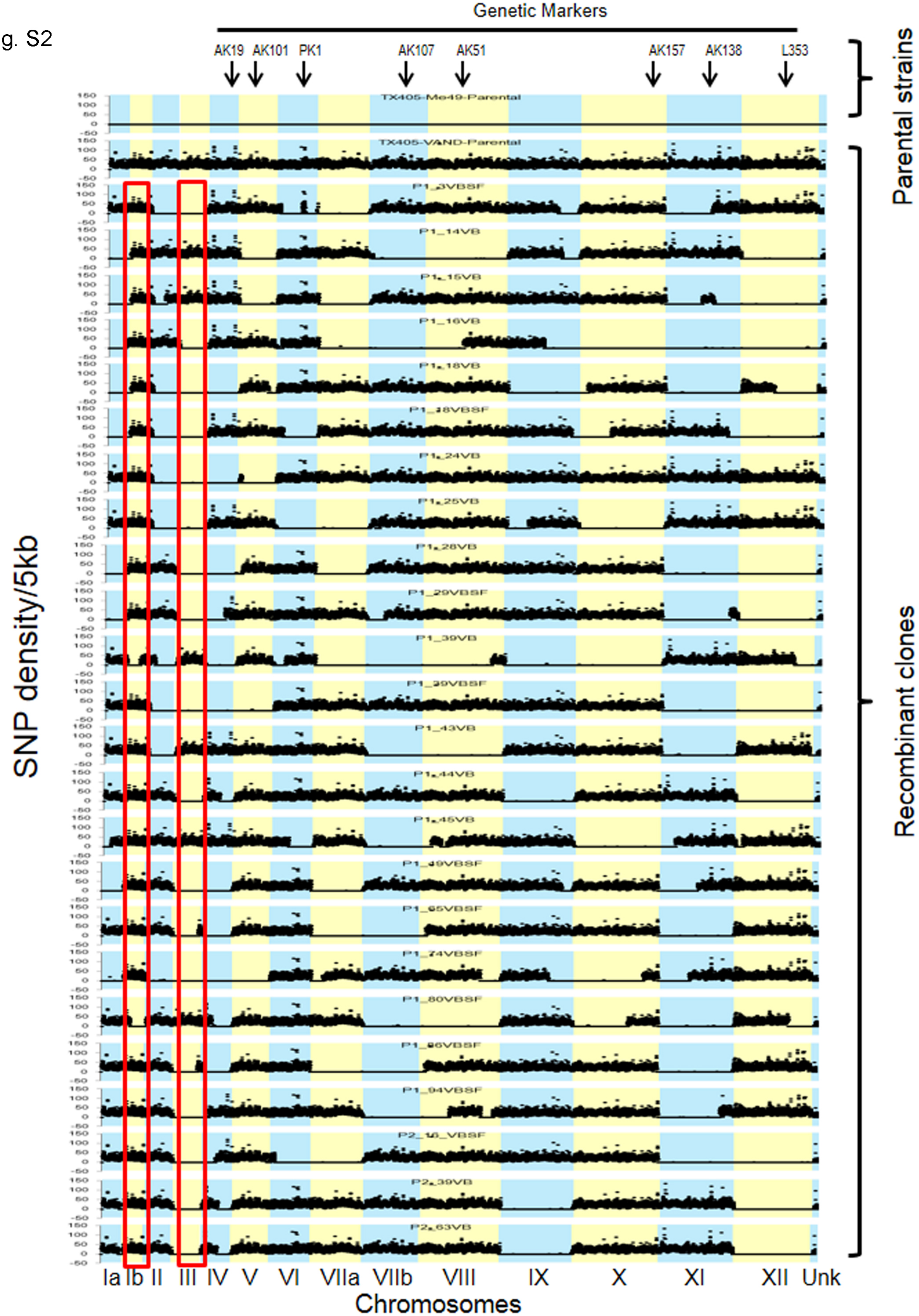

Genetic Markers
AK19
AK101
PK1
AK107
AK51
AK157
AK138
L353
Parental strains
Recombinant clones
TX405-Me49-Parental
TX405-VAND-Parental
P1_3VBSF
P1_14VB
P1_15VB
P1_16VB
P1_18VB
P1_18VBSF
P1_24VB
P1_25VB
P1_28VB
P1_29VBSF
P1_39VB
P1_39VBSF
P1_43VB
P1_44VB
P1_45VB
P1_49VBSF
P1_65VBSF
P1_74VBSF
P1_80VBSF
P1_86VBSF
P1_94VBSF
P2_16_VBSF
P2_39VB
P2_63VB
SNP density/5kb
Ia Ib II III IV V VI VIIa VIIb VIII IX X XI XII Unk
Chromosomes

Fig. S3

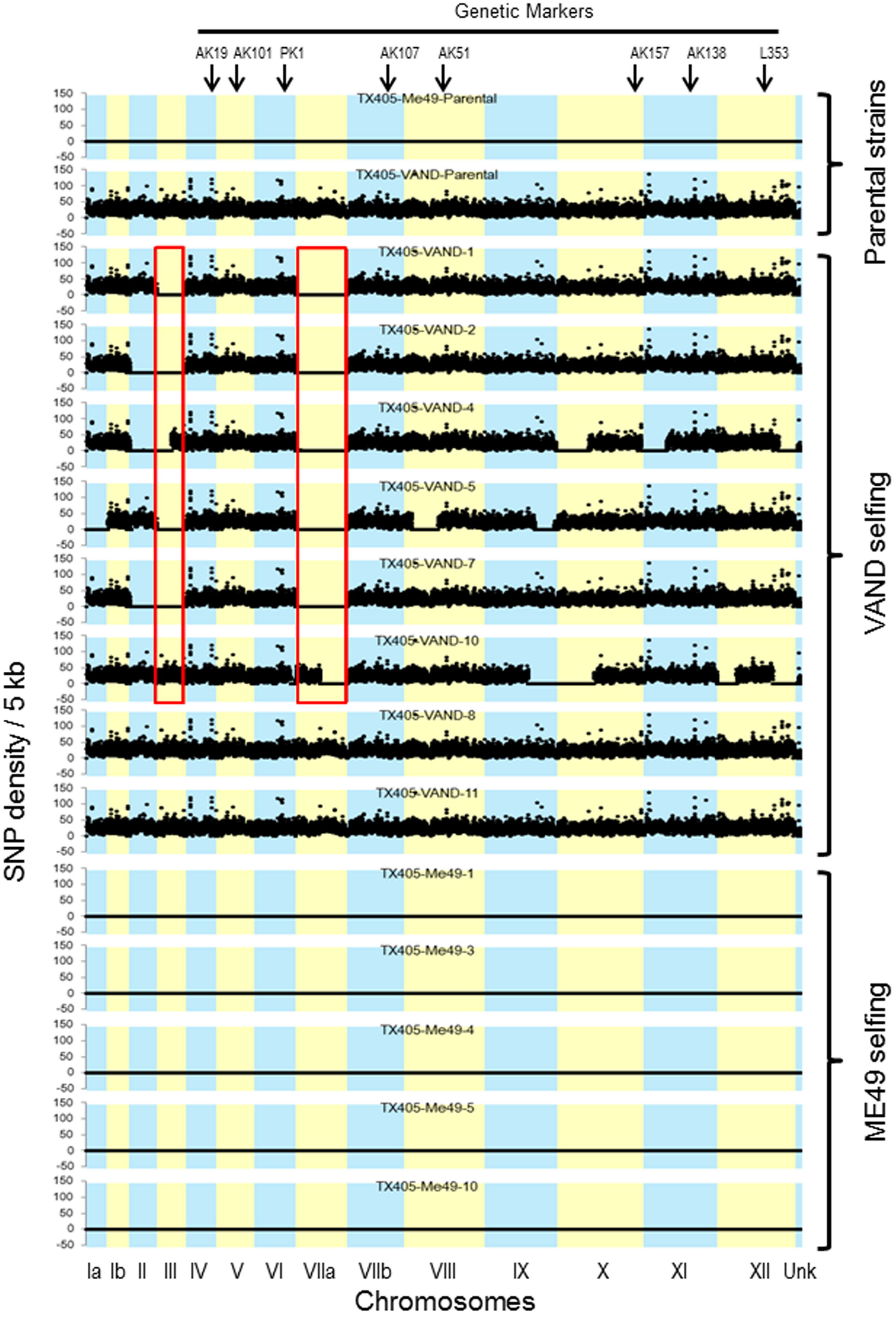

Genetic Markers
AK19
AK101
PK1
AK107
AK51
AK157
AK138
L353
TX405-Me49-Parental
TX405-VAND-Parental
TX405-VAND-1
TX405-VAND-2
TX405-VAND-4
TX405-VAND-5
TX405-VAND-7
TX405-VAND-10
TX405-VAND-8
TX405-VAND-11
TX405-Me49-1
TX405-Me49-3
TX405-Me49-4
TX405-Me49-5
TX405-Me49-10
Parental strains
VAND selfing
ME49 selfing
SNP density / 5 kb
Ia
Ib
II
III
IV
V
VI
VIIa
VIIb
VIII
IX
X
XI
XII
Unk
Chromosomes

Fig. S4

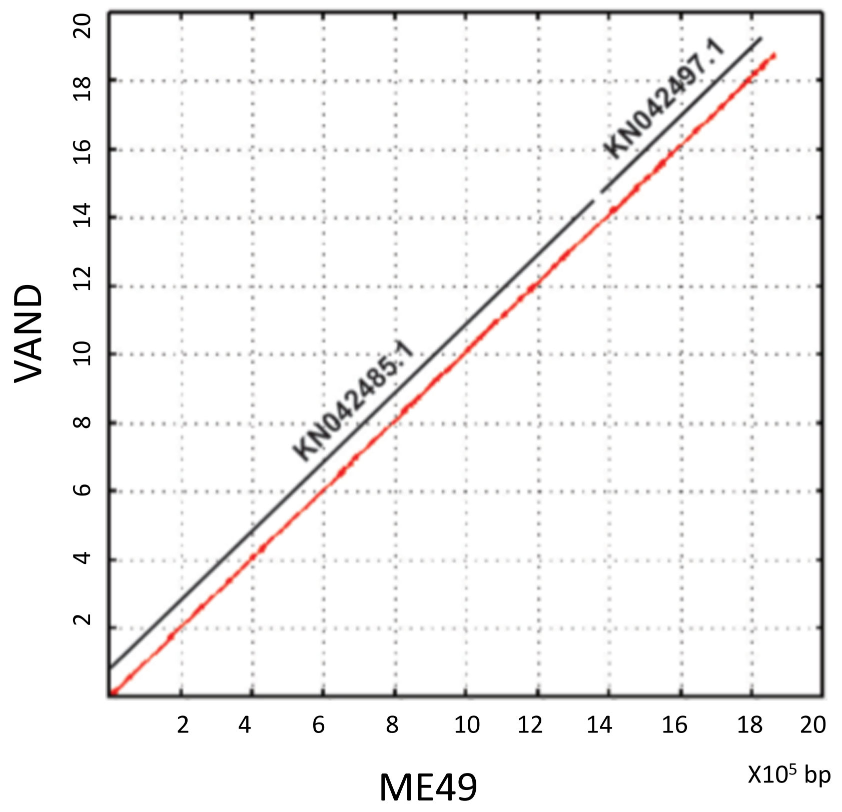

Chromosome 1a comparison
VAND
ME49
X10^5 bp
KN042485.1
KN042497.1
2
4
6
8
10
12
14
16
18
20

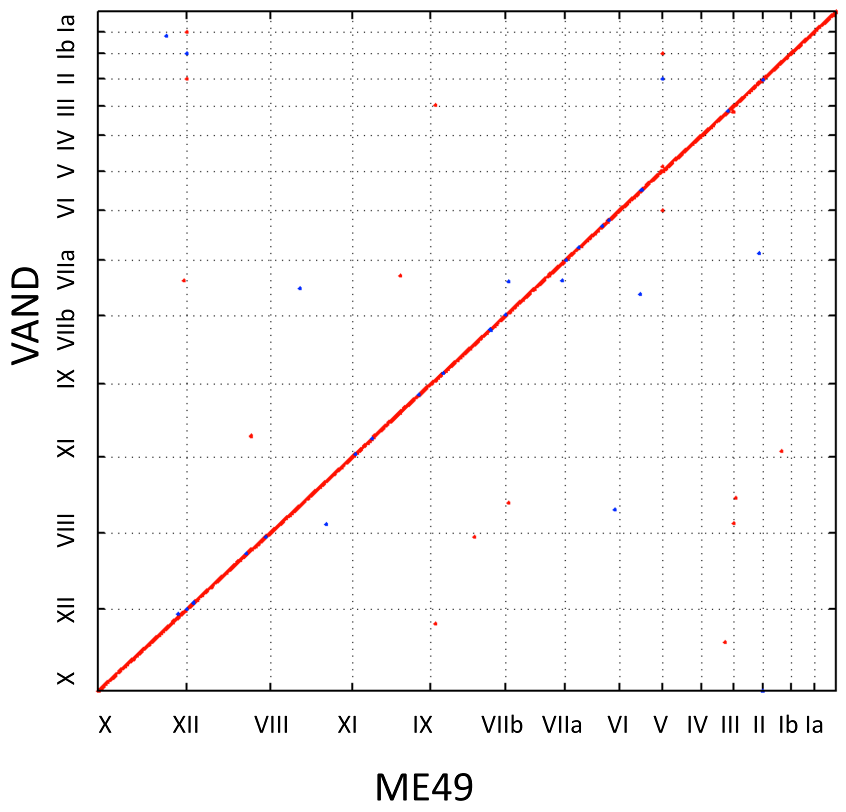

Genome wide comparison
VAND
ME49
X
XII
VIII
XI
IX
VIIb
VIIa
VI
V
IV
III
II
Ib
Ia

Table S1. Recombinant progeny from VAND and ME49 cross used in this study

			Chromosome locaiton and marker name[b]							
			IV	V	VI	VIIB	VIII	X	XI	XII
Number	Recombinant progeny	Clone selection (PCR vs Rx)[a]	AK19	AK101	PK1	AK107	AK51	AK157	AK138	L353
1	ME49 Parental	NA	Red	Red	Red	Red	Red	Red	Red	Red
2	VAND Parental	NA	Green	Green	Green	Green	Green	Green	Green	Green
3	P1_14VB	PCR	Green	Red	Green	Red	Red	Green	Green	Red
4	P1_15VB	PCR	Green	Red	Green	Green	Green	Green	Green	Red
5	P1_16VB	PCR	Green	Green	Green	Red	Green	Red	Red	Red
6	P1_18VB	PCR	Red	Green	Green	Green	Green	Green	Red	Red
7	P1_18VBSF	Rx	Green	Green	Red	Green	Green	Green	Green	Red
8	P1_24VB	PCR	Green	Red	Green	Green	Green	Green	Green	Green
9	P1_25VB	PCR	Green	Green	Red	Green	Green	Red	Green	Green
10	P1_28VB	PCR	Red	Green	Green	Green	Green	Green	Red	Red
11	P1_39VB	PCR	Red	Green	Green	Red	Red	Red	Green	Green
12	P1_39VBSF	Rx	Red	Red	Green	Green	Green	Green	Red	Red
13	P1_3VBSF	Rx	Green	Green	Red	Green	Green	Green	Green	Green
14	P1_43VB	PCR	Red	Green	Green	Red	Red	Green	Red	Green
15	P1_44VB	PCR	Red	Green	Green	Green	Green	Green	Green	Red
16	P1_45VB	PCR	Green	Green	Red	Red	Green	Red	Green	Green
17	P1_49VBSF	Rx	Red	Green	Green	Green	Green	Green	Green	Green
18	P1_65VBSF	Rx	Red	Green	Green	Red	Green	Green	Red	Green
19	P1_74VBSF	Rx	Red	Red	Green	Green	Green	Green	Green	Green
20	P1_80VBSF	Rx	Red	Green	Green	Red	Red	Green	Red	Green
21	P1_86VBSF	Rx	Red	Green	Green	Red	Green	Green	Red	Green
22	P1_94VBSF	Rx	Green	Green	Green	Red	Green	Green	Red	Green
23	P2_16VBSF	Rx	Green	Green	Red	Green	Green	Green	Red	Red
24	P2_39VB	PCR	Red	Green	Green	Green	Green	Green	Green	Red
25	P2_63VB	PCR	Red	Green	Green	Green	Green	Green	Green	Red
26	P2_29VBSF	Rx	Green	Green	Green	Green	Green	Green	Red	Red

Red box indicates ME49 genotype

Green box indicates VAND genotype

[a] Informative clones were selected based on either 10 PCR-RLFP (PCR) markers or SNF/FUDR drug resistance (Rx).

[b] Chomosomal location of each restriction fragment length polymorphism PCR marker as indicated in ToxoDB (www.toxodb.org)

Table S2 Locations and chacateristics of double crossover events

Chromosome	Start	End	Multiple hits[a]	Assemblies[b]	Repeat Masker[c]	2x filter[d]	Uniparental inheritance[e]	ME49toVAND	VANDtoME49	Number of progeny with crossover	gffStart	gffEnd	Annotation	Location[f]
TGME49_chrVIII	6674536	6675294	Yes	tgme49_asmbl.59	No	No	Yes	19	0	19			no annotation	no gene
TGME49_chrIV	123	630	Yes	chrIV-Multiple segm	No	No	Yes	11	0	11			no annotation	no gene
TGME49_chrXII	1999816	1999865	Yes	ABPA02001841,ABP	No	Yes	Yes	11	0	11	1990444	2003898	ID=TGME49_217680;description=hypothetical+protein	intragenic
TGME49_chrII	2277247	2278488	Yes	ABPA02001808,tgm	No	Yes	Yes	9	0	9			no annotation	no gene
TGME49_chrVIIa	2220741	2220949	No	Found itself	No	No	No	3	0	3	2216404	2220785	ID=TGME49_203810;description=kelch+repeat-containing+protein	intragenic
TGME49_chrVIII	6884105	6885584	No	Found itself	Yes	No	No	3	0	3	6883172	6887049	ID=TGME49_200440;description=hypothetical+protein	intragenic
TGME49_chrXI	2571875	2572816	Yes	chrXI-Multiple segm	No	Yes	No	2	0	2	2560227	2573757	ID=TGME49_312280;description=pre-mRNA-splicing+factor+ATP-dependent+	intragenic
TGME49_chrIa	653268	654925	No	Found itself	Yes	No	No	0	1	1	653872	654666	ID=TGME49_294025;description=hypothetical+protein	intragenic
TGME49_chrIa	830820	831401	No	Found itself	No	No	No	1	0	1	816367	840385	ID=TGME49_294350;description=DEAD%2FDEAH+box+helicase+domain-con	intragenic
TGME49_chrIa	909661	909917	No	Found itself	No	No	No	1	0	1	908138	914886	ID=TGME49_294430;description=hypothetical+protein	intragenic
TGME49_chrIb	584924	585578	No	Found itself	No	No	No	1	0	1	576844	587870	ID=TGME49_208070;description=inositol+polyphosphate+kinase	intragenic
TGME49_chrIII	799663	799946	No	Found itself	No	No	No	0	1	1	800581	816753	ID=TGME49_253170;description=zinc+carboxypeptidase%2C+putative	downstream-636bases
TGME49_chrIII	1520268	1521331	Yes	tgme49_asmbl.1164	No	No	No	1	0	1	1519073	1528741	ID=TGME49_254200;description=anticodon+binding+domain-containing+pro	intragenic
TGME49_chrII	1263784	1264263	No	Found itself	No	No	No	1	0	1	1258625	1271648	ID=TGME49_222860;description=eukaryotic+translation+initiation+factor%2	intragenic
TGME49_chrII	1592247	1592678	No	Found itself	No	No	No	1	0	1	1591536	1598773	ID=TGME49_297180;description=hypothetical+protein	intragenic
TGME49_chrIV	1055591	1056146	No	Found itself	No	No	No	0	1	1	1050513	1071691	ID=TGME49_319308;description=hypothetical+protein	intragenic
TGME49_chrIX	532208	532567	No	Found itself	No	No	No	1	0	1			no annotation	no gene
TGME49_chrIX	658436	659222	No	Found itself	No	No	No	1	0	1	656339	662781	ID=TGME49_267430;description=DnaJ+domain-containing+protein	intragenic
TGME49_chrIX	2481570	2481840	No	Found itself	No	No	No	1	0	1	2479431	2481833	ID=TGME49_288370;description=hypothetical+protein	intragenic
TGME49_chrIX	3620635	3621144	No	Found itself	No	No	No	0	1	1	3616271	3626340	ID=TGME49_290170;description=kelch+repeat+domain+containing%2FSerin	intragenic
TGME49_chrVIIa	434388	435285	No	Found itself	Yes	No	No	1	0	1	431978	441797	ID=TGME49_280410;description=3%275%27-cyclic+nucleotide+phosphodies	intragenic
TGME49_chrVIIa	1900899	1901621	No	Found itself	No	No	No	0	1	1	1887392	1909019	ID=TGME49_204310;description=hypothetical+protein	intragenic
TGME49_chrVIIa	3498856	3499248	No	Found itself	No	No	No	1	0	1	3498927	3508284	ID=TGME49_202230;description=histone+deacetylase+HDAC5+%28HDAC5%	intragenic
TGME49_chrVIIa	4233169	4234987	Yes	tgme49_asmbl.1592	Yes	No	No	1	0	1	4229009	4234459	ID=TGME49_281630;description=hydroxyacylglutathione+hydrolase	intragenic
TGME49_chrVIIa	4410156	4410773	No	Found itself	No	No	No	0	1	1	4404312	4414733	ID=TGME49_282150;description=hypothetical+protein	intragenic
TGME49_chrVIIb	958228	958825	No	Found itself	No	No	No	1	0	1	950567	970111	ID=TGME49_262880;description=hypothetical+protein	intragenic
TGME49_chrVIIb	966177	966523	No	Found itself	No	No	No	1	0	1	950567	970111	ID=TGME49_262880;description=hypothetical+protein	intragenic
TGME49_chrVIIb	1261992	1262841	No	Found itself	Yes	No	No	0	1	1	1262306	1273227	ID=TGME49_262420;description=AP2+domain+transcription+factor+APVIIb-	intragenic
TGME49_chrVIIb	1719392	1720164	No	Found itself	No	No	No	1	0	1	1712310	1719667	ID=TGME49_261480;description=phosphatidyl+serine+synthase	intragenic
TGME49_chrVIIb	2994031	2994844	No	Found itself	No	No	No	0	1	1	2994627	2997662	ID=TGME49_259040;description=hypothetical+protein	intragenic
TGME49_chrVIII	147954	148095	No	Found itself	No	No	No	1	0	1	145397	163914	ID=TGME49_229310;description=mediator+complex+subunit+MED14+%28M	intragenic
TGME49_chrVIII	1136335	1136729	No	Found itself	No	No	No	1	0	1	1137557	1149165	ID=TGME49_230960;description=splicing+factor+3b%2C+subunit+3%2C+13	upstream-829bases
TGME49_chrVIII	2638018	2638626	No	Found itself	No	No	No	1	0	1	2639548	2658710	ID=TGME49_233430;description=hypothetical+protein	downstream-923bases
TGME49_chrVIII	2716714	2717562	No	Found itself	No	No	No	1	0	1	2713339	2718359	ID=TGME49_233550;description=polyketide+cyclase%2Fdehydrase	intragenic
TGME49_chrVIII	3945181	3946360	No	Found itself	No	No	No	1	0	1	3944996	3957560	ID=TGME49_272475;description=protein+kinase+domain-containing+protein	intragenic
TGME49_chrVIII	4584149	4584687	No	Found itself	Yes	No	No	1	0	1	4583224	4586361	ID=TGME49_271300;description=DNA-directed+RNA+polymerase+II+RPB7+	intragenic
TGME49_chrVIII	5175014	5176112	No	Found itself	No	No	No	1	0	1	5167606	5177669	ID=TGME49_270580;description=HECT-domain+%28ubiquitin-transferase%2	intragenic
TGME49_chrV	1373609	1375294	No	Found itself	No	No	No	1	0	1	1371223	1376456	ID=TGME49_213840;description=hypothetical+protein	intragenic
TGME49_chrXII	480662	481254	No	Found itself	No	No	No	1	0	1	474441	483460	ID=TGME49_307980;description=GTP-binding+protein+lepA%2C+putative	intragenic
TGME49_chrXII	2131554	2132263	No	Found itself	Yes	No	No	1	0	1	2121442	2133547	ID=TGME49_217790;description=S1+RNA+binding+domain-containing+prote	intragenic
TGME49_chrXII	4145687	4146428	No	Found itself	Yes	No	No	1	0	1	4143912	4157005	ID=TGME49_248750;description=hypothetical+protein	intragenic
TGME49_chrXII	4233999	4234554	No	Found itself	No	No	No	1	0	1	4231788	4235472	ID=TGME49_248870;description=SNARE+associated+Golgi+protein	intragenic
TGME49_chrXII	5802586	5803275	No	Found itself	Yes	No	No	0	1	1	5794929	5811038	ID=TGME49_278870;description=myosin+F	intragenic
TGME49_chrXII	6337738	6338163	No	Found itself	No	No	No	0	1	1	6331322	6337075	ID=TGME49_278050;description=proteasome+subunit+alpha+type+1%2C+p	downstream-664bases
TGME49_chrXII	6425638	6426098	Yes	tgme49_asmbl.1670	Yes	No	No	0	1	1	6406571	6426608	ID=TGME49_277930;description=hypothetical+protein	intragenic
TGME49_chrXII	6489026	6489582	No	Found itself	Yes	No	No	1	0	1	6483139	6490674	ID=TGME49_277840;description=Ras+family+protein	intragenic
TGME49_chrXI	1539543	1540381	No	Found itself	No	No	No	0	1	1	1535663	1546736	ID=TGME49_310720;description=hypothetical+protein	intragenic
TGME49_chrXI	1942988	1943322	No	Found itself	No	No	No	1	0	1	1942384	1946128	ID=TGME49_311280;description=hypothetical+protein	intragenic
TGME49_chrXI	2472933	2473923	No	Found itself	Yes	No	No	0	1	1	2471427	2475160	ID=TGME49_312175;description=hypothetical+protein	intragenic
TGME49_chrXI	3453329	3453765	No	Found itself	No	No	No	0	1	1	3452139	3452645	ID=TGME49_313510;description=hypothetical+protein	upstream-685bases
TGME49_chrXI	3569315	3570210	No	Found itself	No	No	No	0	1	1	3527394	3573580	ID=TGME49_313630;description=hypothetical+protein	intragenic
TGME49_chrXI	5915752	5915972	No	Found itself	No	No	No	1	0	1	5905938	5915009	ID=TGME49_216760;description=RNA+pseudouridine+synthase+superfamily	upstream-744bases
TGME49_chrX	788025	788788	No	Found itself	No	No	No	1	0	1	786659	790796	ID=TGME49_227830;description=mitochondrial+import+inner+membrane+tr	intragenic
TGME49_chrX	1310771	1311025	No	Found itself	No	No	No	0	1	1	1307469	1310816	ID=TGME49_226830;description=DnaK+family+protein	intragenic
TGME49_chrX	2553783	2555054	Yes	tgme49_asmbl.1635	No	No	No	1	0	1	2547288	2553010	ID=TGME49_225060;description=nucleoredoxin+family+protein	upstream-774bases
TGME49_chrX	2606319	2607596	No	Found itself	No	No	No	0	1	1			no annotation	no gene
TGME49_chrX	2733516	2733980	No	Found itself	No	No	No	0	1	1	2727980	2764455	ID=TGME49_224870;description=hypothetical+protein	intragenic
TGME49_chrX	4906668	4906976	No	Found itself	No	No	No	1	0	1	4905534	4916069	ID=TGME49_235490;description=hypothetical+protein	intragenic
TGME49_chrX	5322638	5322966	No	Found itself	No	No	No	1	0	1			no annotation	no gene

a BLASTN versus ME49 reference genome (ToxoxDB), e ≤ 10-4, ≥ 90% identity. Mutiple BLAST hits are indicated as "yes".
b BLAST hits to multiple regions on the same chromosome are reported as "ChrNumber-multiple segments". If secondary BLAST hits are found in new assemblies, the assembly number is reported. Single hits are reported as "found itself".
c Regions identified with Repeat Masker are indicated as "yes"
d Regions containing more than 2X average coverage of reads are indicated as "yes"
e When all progeny inherited the same allele for mutiple events, they are indicated as "yes"
f Annotaions for regions within a gene or within 1,000 bp of a gene are indicated.

Table S3 Confirmation of double crossovers

Chromosome	Position	Size (bp) of double crossover	# Progeny with double crossover	ME49	VAND	Double cross over Progeny	Control Progeny
TGME49_chrVIIa	2219690	548	3[a]	A	G	G	A
TGME49_chrVIIa	2219706			T	C	C	T
TGME49_chrVIIa	2219920			T	C	C	T
TGME49_chrVIIa	2220277			A	G	G	A
TGME49_chrVIIa	2220452			C	A	A	C
TGME49_chrVIIa	2220741			G	A	G	G
TGME49_chrVIIa	2220847			T	C	T	T
TGME49_chrVIIa	2220883			T	G	T	T
TGME49_chrVIIa	2220915			C	T	C	C
TGME49_chrVIIa	2220949			T	C	T	T
TGME49_chrVIIa	2221289			C	T	T	C
TGME49_chrVIIa	2221771			T	C	C	T
TGME49_chrVIIa	2221841			T	C	C	T
TGME49_chrVIIa	2221964			G	C	C	G
TGME49_chrVIIa	2221967			G	A	A	G
TGME49_chrXII	6337004	425	1[b]	T	C	T	C
TGME49_chrXII	6337043			A	G	A	G
TGME49_chrXII	6337342			A	T	A	T
TGME49_chrXII	6337366			T	G	T	G
TGME49_chrXII	6337382			T	G	T	G
TGME49_chrXII	6337494			C	T	T	T
TGME49_chrXII	6337508			A	G	G	G
TGME49_chrXII	6337688			C	G	C	G
TGME49_chrXII	6337738			C	A	A	A
TGME49_chrXII	6337832			C	T	T	T
TGME49_chrXII	6337868			C	T	T	T
TGME49_chrXII	6337884			T	C	C	C
TGME49_chrXII	6337981			G	A	A	A
TGME49_chrXII	6338163			T	C	T	C
TGME49_chrXII	6338191			G	C	G	C
TGME49_chrXII	6338367			G	A	G	A
TGME49_chrXII	6338427			A	G	A	G
TGME49_chrXII	6338429			T	C	T	C
TGME49_chrIb	584044	654	1[c]	G	T	T	T
TGME49_chrIb	584190			C	A	A	A
TGME49_chrIb	584489			C	T	T	T
TGME49_chrIb	584542			A	G	G	G
TGME49_chrIb	584783			G	C	C	C
TGME49_chrIb	584924			G	T	G	T
TGME49_chrIb	584951			A	G	A	G
TGME49_chrIb	585081			T	C	T	C
TGME49_chrIb	585120			C	T	C	T
TGME49_chrIb	585174			T	G	T	G
TGME49_chrIb	585236			C	A	C	A
TGME49_chrIb	585353			C	T	C	T
TGME49_chrIb	585578			A	C	C	C
TGME49_chrIb	585623			A	C	C	C
TGME49_chrIb	585705			G	A	A	A
TGME49_chrIb	585789			A	G	G	G
TGME49_chrIb	585855			A	G	G	G

[a] Double crossover Progeny = P1_44VB, Control Progeny = P1_39VB. Forward primer: 5'CAGACACACTCCCTCCTTTCC3'; Reverse primer: 5'CTGCAACGGTTTCACAGTGC3'

[b] Double crossover Progeny = P1_29VBSF, Control Progeny = P1_3VBSF. Forward primer: 5'CTGGTCGCGAAACGTC3'; Reverse primer: 5'CCAACTGCAGGGGAG3'

[c] Double crossover Progeny = P1_15VB, Control Progeny = P1_14VB. Forward primer: 5'CAGAGGAACGGTCGCACC3'; Reverse primer:
